# One-Pot, Highly Stereoselective Synthesis of Dithioacetal-α,α-Diglycosides

**DOI:** 10.3390/molecules23040914

**Published:** 2018-04-15

**Authors:** Maria F. Céspedes Dávila, Jérémy P. Schneider, Amélie Godard, Damien Hazelard, Philippe Compain

**Affiliations:** Laboratoire d’Innovation Moléculaire et Applications (LIMA), Université de Strasbourg, Université de Haute-Alsace, CNRS (UMR 7042), Equipe Synthèse Organique et Molécules Bioactives (SYBIO), Ecole Européenne de Chimie, Polymères et Matériaux, 25 rue Becquerel, 67000 Strasbourg, France; mafercespedes@hotmail.com (M.F.C.D.); jeremy.schneider@live.fr (J.P.S.); amelie.godard@etu.unistra.fr (A.G.); damien.hazelard@unistra.fr (D.H.)

**Keywords:** glycomimetics, thioglycosides, dithioacetals, 1,6-anhydrosugars, ring-opening

## Abstract

A one-step access to dithioacetal-α,α-diglycosides is reported. The synthetic strategy is based on the thioacetalization of aldehydes or ketones via highly stereoselective ring-opening of 1,6 anhydrosugars with bis(trimethylsilyl)sulfide.

## 1. Introduction

**Scheme 1:**
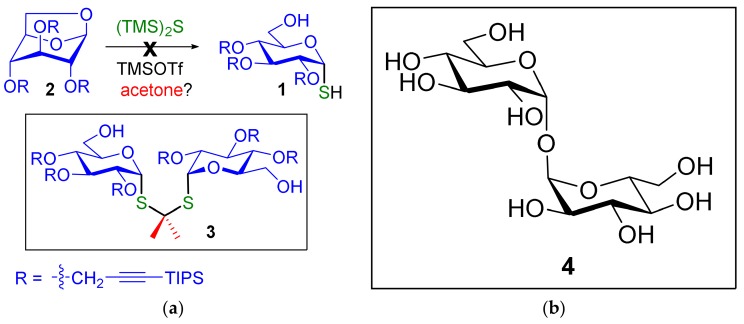
(**a**) Unexpected formation of dithioacetal diglucoside **3**. (**b**) Trehalose structure.

Carbohydrates are a major class of inspiring structures for the design of biologically active compounds. These densely functionalized chiral molecules represent also a prolific source of useful building blocks and molecular tools for asymmetric synthesis. Moreover, their relative structural complexity has been a fertile ground for accidental discoveries in organic synthesis [1]. In this context, we have recently reported a molecular Lego approach for the synthesis of functionalized cyclic and acyclic neo-oligosaccharides [2]. Our strategy was based on the linkage of bifunctional building blocks by Cu(I)-catalyzed azide−alkyne cycloaddition, the reducing end of the sugars being activated as a glycosyl azide and masked as a 1,6-anhydro sugar [2,3]. To explore the interest of thiol-click chemistry for such modular strategies [4,5], we needed to synthesize thioglycoside **1** (Scheme 1). In a first attempt, (TMS)_2_S ring-opening of 1,6-anhydro sugar **2** mediated by TMSOTf following Zhu’s protocol [6] did not lead to the desired α-glucosyl thiol **1** (Scheme 1). Much to our surprise, dithioacetal-α,α-diglucoside **3** was obtained as the major product in low yields and as a single diastereomer. We assumed that the unexpected formation of dithioacetal **3** was due to the accidental presence of traces of acetone in the reaction mixture. Dithioacetal diglycosides have almost no precedent in the literature [7,8]. These compounds may be viewed as analogues of trehalose (**4**), a biologically relevant nonreducing disaccharide found in microorganisms, bacteria, plants and invertebrates [9,10,11]. Examples of related diglycoside acetals designed for asymmetric synthesis, molecular imaging or for their biological interest have been described in the literature [12,13]. Chemistry wise, we were also attracted by the efficiency of this highly stereoselective process in which the formation of multiple C-S single bonds generates an original thiodisaccharide analogue in one step. In this communication, we wish to report our preliminary exploration of the synthetic scope of this TMSOTf-mediated one-pot reaction leading to dithioacetal diglycosides via 1,6-anhydro sugar ring-opening.

## 2. Results and Discussion

### 2.1. Optimization of the Reaction Conditions

**Figure 1:**
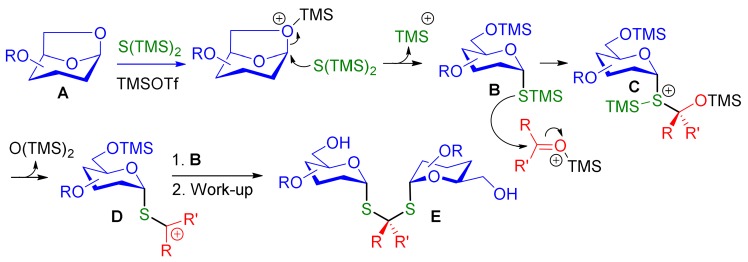
Tentative mechanism for the TMSOTf-mediated formation of dithioacetal diglycosides.

Exploration of the synthetic scope of the one-pot process was first performed with 1,6-anhydro-D-glucose **5a [14]** using benzaldehyde as the model carbonyl partner (Table 1). The choice of benzaldehyde was guided by mechanistic considerations as we anticipated that this non-enolisable carbonyl compound offered a good equilibrium between reactivity, stability and low volatility. According to the tentative mechanism proposed in Figure 1, TMSOTf is expected to play a double role in the formation of the dithioacetal product. In the first step, TMSOTf promotes the highly diastereoselective (TMS)_2_S ring-opening of anhydrosugar **A** following a S_N_2-type mode [6]. In a second step, addition of the resulting α-thioglycoside **B** to the TMSOTf-activated carbonyl compound affords the stabilized α-thio carbocation **D** via thioacetal **C** [15,16,17,18]. The subsequent interception of this carbocation by a second molecule of α-thioglycoside **B** affords the dithioacetal-α,α-diglycoside **E** after aqueous work-up.

According to Zhu’s optimized conditions [6], 1.4 equiv. of (TMS)_2_S were used in refluxing dichloromethane and the quantity of TMSOTf was increased from 0.4 equiv. to stoichiometric quantity to take into account the activation of the carbonyl compound according to our tentative mechanism (Figure 1). The reaction was performed in hermetically sealed tubes to avoid solvent evaporation. First attempts to obtain dithioacetal **6a** by introducing benzaldehyde at the beginning of the reaction led to unidentified degradation products. To avoid possible inhibition of the first step of the process, i.e., the formation of the key thioglycoside intermediate **B**, the carbonyl partner was added subsequently after conversion of anhydrosugar **5a** to the corresponding thioglycoside derivative. After several assays performed in the absence of benzaldehyde, we found that a complete conversion of the starting material into thioglycoside **7** could be achieved after 2 h when 1.1 equiv. of TMSOTf were used. Various experimental parameters were then examined for the second step. In a first attempt made at room temperature using 0.5 equiv. of benzaldehyde, the desired dithioacetal **6a** was obtained in 13% yield, the major product being the unconverted thioglycoside **7** (entry 1, Table 1). To increase the conversion of **5a** to **6a**, the amount of benzaldehyde was doubled and the temperature reduced to avoid possible degradation. Following these conditions, the yield of the one-pot process could be doubled to 27% at −15 °C and increased to 45% at −30 °C (entries 2–3, Table 1). The yield was further improved to 60% by adding 4Å molecular sieves [18] in the reaction mixture to avoid any possible inhibition of the thioacetalization reaction by traces of water in the reaction mixture (entry 4, Table 1). It was anticipated that the presence of water may facilitate the in situ hydrolysis of the glucosyl silyl sulfide intermediate **B** to the corresponding less reactive thiol (Figure 1) [19]. The addition of more equivalents of benzaldehyde at −78 °C in the presence of 4Å molecular sieves led to a clean reaction mixture as judged on TLC. However, no improvement of the reaction yields was observed (entries 5–7, Table 1). 

### 2.2. Reaction Scope

We then tested the one-pot reaction on a series of aldehydes under the optimized conditions to evaluate its synthetic scope (Table 2). The influence of electron-withdrawing and electron-donating substituents on the aromatic aldehyde moiety was explored with benzaldehyde analogues. The presence of a *para*-methoxy group did not improve the efficiency of the process whereas substitution with a trifluoromethyl group was found to strongly decrease the yield of the reaction (entries 2–3). We were pleased to see that the one-pot synthesis of dithioacetal glycosides could be performed with enolizable aliphatic aldehydes, however in lower yields compared to benzaldehyde (entries 4–5). Not surprisingly, the modest yields obtained with pivaladehyde compared to linear aldehydes suggested that the thioacetalization process was sensitive to steric hindrance (entry 6). The experimental conditions were found to be compatible with allyl protecting groups (entry 7). 

**Scheme 2:**
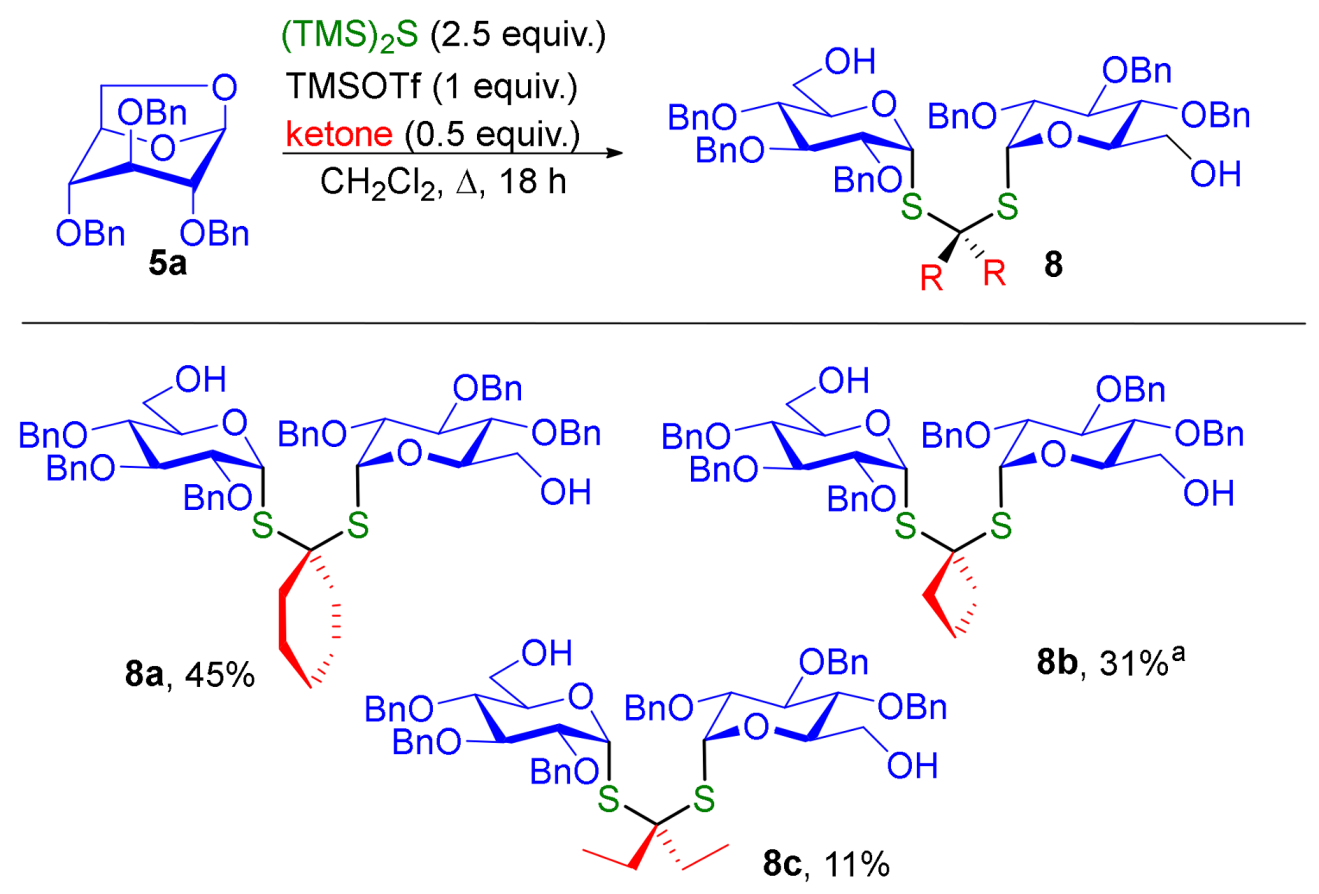
Scope of ketones in the one-pot formation of dithioketal-α,α-diglycosides.

The one-pot synthesis of dithioketal-α,α-diglycosides were explored with cyclic and acyclic ketones (Scheme 2). The expected lowest reactivity of ketones compared to aldehydes resulted in lower yields. Application of optimal conditions for the synthesis of **6** afforded acceptable yields only for cyclobutanone giving **8b** in 31% yield. Under these conditions, no desired products or poor conversions were obtained with cyclohexanone and 3-pentanone. After extensive optimization with cyclohexanone we founded that large excess of TMS_2_S (2.5 equiv.) is required and that the cyclohexanone have to be present from the beginning of the reaction. With these conditions we are delighted to obtain compound **8a** with 45% yield. The application of these conditions with 3-pentanone afforded **8c** in poor yield showing the less reactivity of acyclic ketone for this one-pot process.

It is noteworthy that the ^1^H NMR spectra of dithioketal glycosides **8** obtained from ketones are much simpler than those generated from aldehydes. In **8**, the two sugars moieties are magnetically equivalent. One signal is thus observed for the two anomeric protons which appear as a doublet with a vicinal coupling constant of ~5 to 6 Hz. In contrast, two distinct doublets are observed for the anomeric protons in **6** (*J* ~ 5 to 6 Hz). In the case of compounds **6**, the two pyranose moieties are not magnetically equivalent. Tietze et al. have reported a similar observation for related diglycosides acetals [12].

## 3. Materials and Methods

### 3.1. Preparation of Anhydroglucoses **2** and **5**

*1,6-Anhydro-2,3,4-tri-O-(triisopropylsilylpropargyl)-β-D-glucopyranose* (**2**). To a solution of 1,6-Anhydro-2,3,4-tri-*O*-propargyl-*β*-D-glucopyranose [3] (1 equiv., 675 mg, 2.44 mmol) in THF (60.9 mL) at −70 °C was added LiHMDS (4 equiv., 1 M, 9.77 mL, 9.77 mmol) dropwise. Then after 20 min TIPSCl (4 equiv., 1.94 g, 2.16 mL, 9.77 mmol) was added dropwise. The reaction mixture was stirred for 2 h. The reaction was quenched with MeOH and the mixture was evaporated. Then saturated aqueous solution of NH_4_Cl (40 mL) and Et_2_O (40 mL) were added. The aqueous layer was extracted with Et_2_O (2 × 40 mL). The combined organic extracts were washed with brine (40 mL), dried with Na_2_SO_4_, filtered and concentrated. The crude was purified by flash chromatography (Petroleum Ether/EtOAc 100/0 to 70/30), to afford **2** (1.15 g, 63%) as a yellow solid. ${\left[\alpha \right]}_{D}^{20}$ − 30 (*c* 1, EtOH). IR 2172 (C≡C, weak, sharp) cm^−1^. ^1^H NMR (400 MHz, CDCl_3_) *δ* (ppm) 5.51 (s, 1H, H-1), 4,67 (d, *J* = 5.0 Hz, 1H, H-5,) 4,47–4.25 (m, 6H, OCH_2_C≡CTIPS), 3.97 (dd, *J* = 7.2, 0.8 Hz, 1H, H-6), 3.83 (m, 1H, H-3), 3.74–3.67 (m, 2H, H-4, H-6), 3.60 (s, 1H, H-2), 1.01–1.10 (m, 63H, TIPS). ^13^C NMR (100 MHz, CDCl_3_) *δ* (ppm) 103.0, 102.9, 102.8 (3xC-10), 100.4 (C-1), 88.8, 88.6 (3xC-11), 75.1, 74.7, 74.3, 74.1 (4xCH-O), 65.4 (C-6), 58.0, 57.8, 57.5 (3xOCH_2_C≡CTIPS), 18.7 (CH_3_-CH-Si), 11.3 (CH_3_-CH-Si). HRMS (ESI) *m*/*z* [M + Na]^+^ calculated for [C_42_H_76_O_5_Si_3_Na]^+^: 767.489; found 767.487.

*1,6-Anhydro-2,3,4-tri-O-benzyl-β-D-glucopyranose* (**5a**). To a solution of levoglucosan (1 equiv., 5 g, 30.5 mmol) in DMF (200 mL) at 0 °C was added benzyl bromide (3.5 equiv., 18.3 g, 12.8 mL, 106 mmol). Sodium hydride (60% in oil, 5 equiv., 6.11 g, 152 mmol) was added portionwise. The ice bath was removed, and the reaction was stirred overnight at r.t. Methanol (60 mL) was added, and 15 min later water was added (120 mL). The aqueous layer was extracted with EtOAc (3 × 200 mL), and the combined organic extracts were washed with brine (1 × 200 mL). The organic solution was dried over sodium sulfate, filtered, concentrated to an oil that was purified by flash chromatography (Petroleum Ether/EtOAc 8/2 to 6/4), to afford a solid that was then recrystallized from ethanol to afford **5a** (11.34 g, 26.23 mmol) as white crystals in 86% yield. ^1^H NMR (300 MHz, CDCl_3_) *δ* (ppm) 7.35–7.22 (m, 15H, Ph), 5.46 (s, 1H, H-1), 4.64–4.51 (m, 5H, H-5, O-CH_2_-Ph), 4.45 (d, *J* = 12.1 Hz, 1H, O-CH_2_-Ph) 4.41 (d, *J* = 12.1 Hz, 1H, O-CH_2_-Ph), 3.91 (d, *J* = 7.2 Hz, 1H, H-6), 3.68 (t, *J* = 6.5 Hz, 1H, H-6), 3.59 (m, 1H, H-3), 3.35 (m, 2H, H-2, H-4). MS (ESI): *m*/*z* [M + K]^+^ calculated for [C_27_H_28_O_5_K]^+^: 471.16, found 471.16. The analyses are in good agreement with the experimental data reported in literature [20].

*1,6-Anhydro-2,3,4-tri-O-allyl-β-D-glucopyranose* (**5b**). To a solution of levoglucosan (1 equiv., 1 g, 6.11 mmol) in DMF (45 mL) at −20 °C was added sodium hydride (60% in oil, 4 equiv., 0.98 g, 24.4 mmol) portionwise. The cooling bath was allowed to reach 0 °C within 1 h, then it was cooled again to −20 °C and allyl bromide (4.5 equiv., 2.4 mL, 27.6 mmol) was added under vigorous stirring. The reaction mixture was stirred overnight at r.t. The mixture was diluted with water and extracted with diethyl ether (3 × 100 mL). The organic phases are combined and washed 5 times with water, dried with Na_2_SO_4_, filtered, and concentrated to afford a yellow oil which was purified by flash chromatography (Petroleum Ether/EtOAc, 9/1 to 1/1), to give **5b** (1.63 g, 5.77 mmol) as a yellow oil in 95% yield. ^1^H NMR (400 MHz, CDCl_3_) *δ* (ppm) 5.92–5.79 (m, 3H, OCH_2_CHCH_2_), 5.37 (s, 1H, H-1), 5.26–5.12 (m, 6H, OCH_2_CHCH_2_), 4.51 (m, 1H, H-5), 4.08–4.02 (m, 6H, OCH_2_CHCH_2_), 3.85 (dd, *J* = 7.1, 0.8 Hz, 1H, H-6), 3.64 (dd, *J* = 6.8, 6.0 Hz, 1H, H-6), 3.45 (m, 1H, H-3), 3.24 (m, 1H, H-4), 3.21 (m, 1H, H-2). The analyses are in good agreement with the experimental data reported in literature [21].

### 3.2. General Procedure for the Synthesis of Diglycoside Thioacetals **6**

Molecular sieves 4Å (80 mg) were added to a tube. A solution of anhydroglucose **5** (1 equiv., 0.233 mmol) in CH_2_Cl_2_ (1 mL) was added via cannula to the flask. Bis (trimethylsilyl) sulfide (1.4 equiv., 58.3 mg, 0.0613 mL, 0.327 mmol) followed by trimethylsilyl trifluoromethane sulfonate (1.1 equiv., 57 mg, 0.0466 mL, 0.257 mmol) were added to the mixture. The tube was sealed and the medium was stirred at 60 °C. After 2 h of reaction, a solution of the corresponding aldehyde (1 equiv., 0.233 mmol) in 0.3 mL of CH_2_Cl_2_ was added at −30 °C. The reaction was stirred for 1 h 30 min. The mixture was warmed up to r.t. and washed with saturated aqueous NaHCO_3_ (50 mL). The aqueous layer was extracted with CH_2_Cl_2_ (3 × 50 mL). The organic layers were combined and washed with brine (50 mL), dried over sodium sulfate and concentrated under reduced pressure. The crude obtained was purified by flash column chromatography (Petroleum Ether/EtOAc) to afford compounds **6**.

Dithioacetal **6a**. According to the general procedure for the synthesis of diglycoside thioketals **6**, compound **6a** was obtained as a solid in 60% yield after flash column chromatography (Petroleum Ether/EtOAc, 8/2 to 4/6). ${\left[\alpha \right]}_{D}^{20}$ + 177 (*c* 0.7, CHCl_3_). IR 3431 cm^-1^ (broad O-H). ^1^H NMR (400 MHz, CDCl_3_) *δ* (ppm) 7.44–7.22 (m, 35H, Ph), 5.95 (d, *J* = 5.5 Hz, 1H, H-1 or H-1′), 5.02 (s, 1H, H-7), 5.00–4.97 (m, 2H, O-CH_2_-Ph, H-1 or H-1′), 4.91–4.77 (m, 5H, O-CH_2_-Ph), 4.71 (d, *J* = 10.8 Hz, 1H, O-CH_2_-Ph), 4.62 (d, *J* = 11.4 Hz, 1H, O-CH_2_-Ph), 4.57 (d, *J* = 5.9 Hz, 1H, O-CH_2_-Ph), 4.54 (d, *J* = 5.7 Hz, 1H, O-CH_2_-Ph), 4.38 (d, *J* = 11.5 Hz, 1H, O-CH_2_-Ph), 4.32 (d, *J* = 11.5 Hz, 1H, O-CH_2_-Ph), 4.21 (m, 1H, H-5 or H-5′), 4.14 (m, 1H, H-5 or H-5′), 3.98–3.80 (m, 5H, H-2 or H-2′, H-6, H-6′, H3, H3′), 3.67 (dd, *J* = 9.5; 5.6 Hz, 1H, H-2 or H-2′), 3.63–3.55 (m, 2H, H-6, H6′), 3.37 (dd, *J* = 10.0; 8.8 Hz, 1H, H-4 or H-4′), 3.28 (dd, *J* = 9.9; 8.8 Hz, 1H, H-4 or H-4′). ^13^C NMR (100 MHz, CDCl_3_) *δ* (ppm) 139.4 (Cq-Ar), 138.6 (Cq-Ar), 138.55 (Cq-Ar), 138.1 (Cq-Ar), 137.8 (Cq-Ar), 137.7 (Cq-Ar), 137.3 (Cq-Ar), 128.9–127.8 (35 × CHAr) 82.8 (CH) 82.7 (CH), 82.6 (CH), 81.9 (C-1 or C-1′), 79.4 (CH), 78.8 (CH), 77.8 (C-4 or C-4′), 78.4 (C-4 or C-4′), 75.9 (O-CH_2_-Ph), 75.6 (O-CH_2_-Ph), 75.2 (O-CH_2_-Ph), 72.8 (C5 or C-5′), 72.6 (C-5 or C5′), 72.3 (O-CH_2_-Ph), 71.9 (O-CH_2_-Ph), 63.0 (C-6 or C-6′), 62.2 (C-6 or C-6′), 46.2 (C-7). HRMS (ESI) *m*/*z* [M + K]^+^ calculated for [C_61_H_64_O_10_S_2_K]^+^: 1059.357, found: 1059.366.

Dithioacetal **6b**. According to the general procedure for the synthesis of diglycoside thioketals **6**, compound **6b** was obtained in 16% yield after flash column chromatography (Petroleum Ether/EtOAc, 8/2 to 0/1). ${\left[\alpha \right]}_{D}^{20}$ + 152 (*c* 0.9, CHCl_3_). IR 3428 cm^−1^ (broad O-H). ^1^H NMR (400 MHz, CDCl_3_) *δ* (ppm) 7.69 (*J* = 8.2 Hz, 2H, H-10), 7.62 (*J* = 8.1 Hz, 2H, H-9), 7.45–7.09 (m, 30H, Ph), 5.93 (d, *J* = 5.5 Hz, 1H, H-1 or H-1′), 5.06 (s, 1H, H-7), 4.99 (d, *J* = 10.9 Hz, 1H, O-CH_2_-Ph), 4.91–4.78 (m, 6H, O-CH_2_-Ph, H-1 or H-1′), 4.71 (d, *J* = 10.9 Hz, 1H, O-CH_2_-Ph), 4.63 (d, *J* = 11.2 Hz, 1H, O-CH_2_-Ph), 4.55 (d, *J* = 10.5 Hz, 2H, O-CH_2_-Ph), 4.43 (d, *J* = 11.8 Hz, 1H, O-CH_2_-Ph), 4.39 (d, *J* = 11.7 Hz, 1H, O-CH_2_-Ph), 4.17 (ddd, *J* = 9.9, 7.4, 2.0 Hz, 1H, H-5 or H-5′), 4.09 (ddd, *J* = 9.7, 6.9, 2.8 Hz, 1H, H-5 or H-5′), 3.97–3.76 (m, 5H, H-6, H-6′, H-3, H-3′, H-2 or H-2′) 3.68 (dd, *J* = 9.4, 5.6 Hz, 1H, H-2 or H-2′), 3.64–3.54 (m, 2H, H-6, H-6′) 3.35 (dd, *J* = 10.0; 8.9 Hz, 1H, H-4 or H-4′), 3.28 (dd, *J* = 10.0; 8.8 Hz, 1H, H-4 or H-4′). ^13^C NMR (100 MHz, CDCl_3_) *δ* (ppm) 143.7 (C-8), 138.55 (Cq-Ar), 138.5 (Cq-Ar), 138.0 (Cq-Ar), 137.7 (Cq-Ar), 137.5 (Cq-Ar), 137.2 (Cq-Ar), 128.8-127.8 (34 × CHAr), 125.9 (C9, q, *J* C-F = 4 Hz), 82.65 (CH) 82.6 (CH), 82.55 (CH), 82.0 (CH, C-1 or C-1′), 79.4 (CH), 79.0 (C-2 or C-2′), 78.4 (C-4 or C-4′), 77.7 (C-4 or C-4′), 75.9 (O-CH_2_-Ph), 75.6 (O-CH_2_-Ph), 75.2 (O-CH_2_-Ph), 72.85 (C-5 or C-5′), 72.8 (C-5 or C-5′), 72.5 (O-CH_2_-Ph), 72.4 (O-CH_2_-Ph), 63.1 (C-6 or C-6), 62.1 (C-6 or C-6′), 45.2 (C-7). ^19^F NMR (376 MHz, CDCl_3_) δ (ppm) −66.5 (3F, s, CF_3_). HRMS (ESI) *m*/*z* [M + K]^+^ calculated for [C_62_H_63_F_1_O_10_S_2_K]^+^: 1127.345, found: 1127.332.

Dithioacetal **6c.** According to the general procedure for the synthesis of diglycoside thioketals **6**, compound **6c** was obtained in 45% yield after flash column chromatography (Petroleum Ether/EtOAc, 8/2 to 0/1). ${\left[\alpha \right]}_{D}^{20}$ + 151 (*c* 1.1, CHCl_3_). IR 3437 cm^−1^ (broad O-H). ^1^H NMR (400 MHz, CDCl_3_) *δ* (ppm) 7.49 (d, *J* = 8.8 Hz, 2H, H-10), 7.44–7.37 (m, 2H, Ph), 7.37–7.19 (m, 26H, Ph), 7.18–7.11 (m, 2H, Ph), 6.88 (*J* = 8.8 Hz, 2H, H-9), 5.93 (d, *J* = 5.4 Hz, 1H, H-1 or H-1′), 5.01–4.95 (m, 3H, H-7, H-1 or H-1′, O-CH_2_-Ph), 4.90–4.63 (m, 3H, O-CH_2_-Ph), 4.81 (d, *J* = 5.0 Hz, 2H, O-CH_2_-Ph), 4.78 (d, *J* = 4.9 Hz, 1H, O-CH_2_-Ph), 4.69 (d, *J* = 10.8 Hz, 1H, O-CH_2_-Ph), 4.60 (d, *J* = 11.4 Hz, 1H, O-CH_2_-Ph), 4.55 (d, *J* = 5.4 Hz, 1H, O-CH_2_-Ph), 4.53 (d, *J* = 5.1 Hz, 1H, O-CH_2_-Ph), 4.39 (d, *J* = 11.5 Hz, 1H, O-CH_2_-Ph), 4.32 (d, *J* = 11.6 Hz, 1H, O-CH_2_-Ph), 4.20 (ddd, *J* = 10.1, 7.4, 2.5 Hz, 1H, H-5 or H-5′), 4.13 (ddd, *J* = 9.7, 6.9, 2.8 Hz, 1H, H-5 or H-5′), 3.96–3.79 (m, 5H, H-6, H-6′, H-3, H-3′, H-2 or H-2′), 3.80 (s, 3H, OCH_3_), 3.67 (dd, *J* = 9.5; 5.6 Hz, 1H, H-2 or H-2′), 3.64-3.54 (m, 2H, H-6, H-6′) 3.35 (dd, *J* = 9.8; 8.9 Hz, 1H, H-4 or H-4′), 3.27 (dd, *J* = 10.0; 8.9 Hz, 1H, H-4 or H-4′) ^13^C NMR (100 MHz, CDCl_3_) *δ* (ppm) 159.7 (C-11), 138.65 (Cq-Ar), 138.6 (Cq-Ar), 138.1 (Cq-Ar), 137.8 (Cq-Ar), 137.7 (Cq-Ar), 137.4 (Cq-Ar), 131.3 (C-8), 129.5 (C10), 128.7–127.7 (34 x CHAr), 114.2 (C-9), 82.75 (CH) 82.7 (CH), 82.6 (CH), 81.9 (C-1 or C-1′), 79.4 (CH), 78.9 (C-2 or C-2′), 78.4 (C-4 or C-4′), 77.8 (C-4 or C-4′), 75.9 (O-CH_2_-Ph), 75.6 (O-CH_2_-Ph), 75.1 (O-CH_2_-Ph), 72.7 (C-5 or C-5′), 72.5 (C-5 or C-5′), 72.2 (O-CH_2_-Ph), 71.9 (O-CH_2_-Ph), 63.0 (C-6 or C-6′), 62.1 (C-6 or C-6′), 55.5 (OCH_3_), 45.7 (C-7). HRMS (ESI) *m*/*z* [M + K]^+^ calculated for [C_62_H_66_O_11_S_2_K]^+^: 1089.368, found: 1089.367.

Dithioacetal **6d.** According to the general procedure for the synthesis of diglycoside thioketals **6**, compound **6d** was obtained in 30% yield after flash column chromatography (Petroleum Ether/EtOAc, 8/2 to 4/6). ${\left[\alpha \right]}_{D}^{20}$ + 136 (*c* 1, CHCl_3_). IR 3440 cm^−1^ (broad O-H). ^1^H NMR (400 MHz, CDCl_3_) *δ* (ppm) 7.35–7.26 (m, 30H, Ph), 5.85 (d, *J* = 4.5 Hz, 1H, H-1 or H-1′), 5.37 (d, *J* = 5.4 Hz, 1H, H-1 or H-1′), 5.01–4.95 (m, 2H, O-CH_2_-Ph), 4.93 (s, 1H, O-CH_2_-Ph), 4.90–4.85 (m, 2H, O-CH_2_-Ph), 4.84 (s, 1H, O-CH_2_-Ph), 4.79 (t, *J* = 11.1 Hz, 2H, O-CH_2_-Ph), 4.74 (m, 1H, O-CH_2_-Ph), 4.71–4.70 (m, 2H, O-CH_2_-Ph), 4.63–4.55 (m, 5H, O-CH_2_-Ph), 4.16–4.09 (m, 1H, H5 or H-5′), 4.05–3.95 (m, 2H, H-7, H-4 or H5′), 3.91–3.75 (m, 6H, H-6, H-6′, H-3, H-3′, H-2, H2′), 3.68–3.5 (m, 2H, H-6, H6′), 3.47–3.41 (m, 1H, H-4 or H-4′), 3.36 (dd, *J* = 10.1; 8.4 Hz, 1H, H-4 or H-4′), 1.93–1.87 (m, 2H, H-8), 1.76–1.64 (m, 1H, H-9), 1.50–1.39 (m, 1H, H-9), 0.96–0.92 (m, 3H, H-10). ^13^C NMR (100 MHz, CDCl_3_) *δ* (ppm) 138.8 (Cq-Ar), 138.6 (Cq-Ar), 138.1 (Cq-Ar), 137.8 (Cq-Ar), 137.7 (Cq-Ar), 137.65 (Cq-Ar),128.7-128.0 (30 × CHAr), 82.8 (CH), 82.52 (CH), 82.5 (CH), 81.3 (C-1 or C-1′), 79.6 (CH), 79.3 (CH), 78.4 (C-4 or C4′), 77.4 (C4 or C-4′), 75.9 (CO-CH_2_-Ph), 75.8 (O-CH_2_-Ph), 75.6 (O-CH_2_-Ph), 75.4 (O-CH_2_-Ph), 72.9 (C5 or C5′), 72.5 (O-CH_2_-Ph), 72.3 (C5 or C5′), 72.2 (O-CH_2_-Ph), 62.8 (C-6 or C6′), 61.9 (C6 or C-6′), 45.0 (C-7), 38.5 (C-8), 20.5 (C-9), 13.9 (C-10). HRMS (ESI) *m*/*z* [M + K]^+^ calculated for [C_59_H_68_O_10_S_2_Na]^+^: 1023.415, found: 1023.420.

Dithioacetal **6e.** According to the general procedure for the synthesis of diglycoside thioketals **6**, compound **6e** was obtained in 33% yield after flash column chromatography (Petroleum Ether/EtOAc, 8/2 to 0/1). ${\left[\alpha \right]}_{D}^{20}$ + 173 (*c* 0.95, CHCl_3_). IR 3452 cm^-1^ (broad O-H). ^1^H NMR (400 MHz, CDCl_3_) *δ* (ppm) 7.45–7.17 (m, 30H, Ph), 5.85 (d, *J* = 4.3 Hz, 1H, H-1 or H-1′), 5.38 (d, *J* = 5.3 Hz, 1H, H-1 or H-1′), 5.02–4.54 (m, 12H, O-CH_2_-Ph), 4.11 (ddd, *J* = 10.1, 6.8, 2.5 Hz, 1H, H-5 or H-5′), 4.05–3.93 (m, 2H, H-5 or H-5′, H-7), 3.91–3.75 (m, 6H, H-6, H-6′, H-3, H-3′, H-2, H2′), 3.66 (dd, *J* = 12.6; 5.8 Hz, 1H, H-6 or H-6′), 3.59 (dd, *J* = 11.6, 6.6 Hz, 1H, H-6 or H-6′), 3.48–3.42 (m, 1H, H-4 or H-4′), 3.36 (dd, *J* = 9.9; 8.6 Hz, 1H, H-4 or H-4′), 1.96–1.88 (m, 2H, H-8), 1.76–1.61 (m, 1H, H-9), 1.46–1.36 (m, 1H, H-9), 1.36–1.25 (m, 4H, H-10, H11), 0.97–0.85 (m, 3H, H-12). ^13^C NMR (100 MHz, CDCl_3_) *δ* (ppm) 138.8 (Cq-Ar), 138.6 (Cq-Ar), 138.1 (Cq-Ar), 137.8 (Cq-Ar), 137.7 (Cq-Ar), 137.6 (Cq-Ar), 128.7-127.7 (30 × CHAr), 82.8 (CH), 82.55 (CH), 82.5 (CH), 81.4 (C-1 or C-1′), 79.5 (CH), 79.3 (CH), 78.4 (C4 or C-4′), 77.4 (C-4 or C4′), 75.9 (O-CH_2_-Ph), 75.8 (O-CH_2_-Ph), 75.6 (O-CH_2_-Ph), 75.4 (O-CH_2_-Ph), 72.8 (O-CH_2_-Ph), 72.5 (C-5 or C-5′), 72.3 (C-5 or C-5′), 72.25 (O-CH_2_-Ph), 62.8 (C-6 or C-6′), 61.9 (C-6 or C-6′), 45.4 (C-7), 36.3 (C-8), 31.7 (C-10 or C-11), 26.7 (C-9), 22.7 (C-10 or C-11), 14.2 (C-12). HRMS (ESI) *m*/*z* [M + K]^+^ calculated for [C_60_H_70_O_10_S_2_K]^+^: 1053.404, found: 1053.391.

Dithioacetal **6f.** According to the general procedure for the synthesis of diglycoside thioketals **6**, compound **6f** was obtained as a solid in 25% yield after flash column chromatography (Petroleum Ether/EtOAc, 8/2 to 4/6. ${\left[\alpha \right]}_{D}^{20}$ + 128 (*c* 1.3, CHCl_3_). IR 3445 cm^−1^ (broad O-H). ^1^H NMR (400 MHz, CDCl_3_) *δ* (ppm) 7.39–7.29 (m, 30H, Ph), 5.76 (d, *J* = 4.8 Hz, 1H, H-1 or H-1′), 5.38 (d, *J* = 5.4 Hz, 1H, H-1 or H-1′), 5.01–4.66 (m, 9H, O-CH_2_-Ph), 4.61 (m, 2H, O-CH_2_-Ph), 4.63–4.56 (m, 1H, O-CH_2_-Ph), 4.10–4.05 (m, 1H, H-5 or H-5′), 4.03–3.98 (m, 1H, H-5 or H-5′), 3.90–3.77 (m, 6H, H-7, H-3, H-3′, H6, H6′, H-2 or H-2′), 3.76 (dd, *J* = 9.5; 5.4 Hz, 1H, H-2 or H-2′), 3.66 (dd, *J* = 12.3; 5.9 Hz, 1H, H-6 or H-6′), 3.59 (dd, *J* = 11.6, 6.2 Hz, 1H, H-6 or H-6′), 3.44 (t, *J* = 9.3 Hz, 1H, H-4 or H-4′), 3.36 (t, *J* = 9.2 Hz, 1H, H-4 or H-4′), 1.19 (s, 9H, H-9). ^13^C NMR (100 MHz, CDCl_3_) *δ* (ppm) 138.9 (Cq-Ar), 138.5 (Cq-Ar), 138.0 (Cq-Ar), 137.9 (Cq-Ar), 137.6 (Cq-Ar), 137.5 (Cq-Ar), 128.7-127.7, (30xCHAr), 86.0 (CH, C-1′), 83.0 (CH), 82.0 (CH), 81.3 (CH, C-1), 79.8 (C2′), 79.0 (CH), 78.4 (C4), 77.7 (C4′) 75.8 (O-CH_2_-Ph), 75.6 (O-CH_2_-Ph), 75.4 (O-CH_2_-Ph), 73.8 (O-CH_2_-Ph), 73.0 (C-5), 72.2 (O-CH_2_-Ph), 72.1 (C-5) 62.8 (C-6′), 62.2 (C-6), 59.6 (C-7), 38.6 (C-8), 28.3 (C-9). HRMS (ESI) *m*/*z* [M + Na]^+^ calculated for [C_58_H_66_O_10_S_2_Na]^+^: 1009.399, found: 1009.404.

Dithioacetal **6g.** According to the general procedure for the synthesis of diglycoside thioketals **6**, compound **6g** was obtained in 50% yield after flash column chromatography (Petroleum Ether/EtOAc, 9/1 to 1/9). ${\left[\alpha \right]}_{D}^{20}$ + 388 (c 0.51, CHCl_3_). IR 3425 cm^−1^ (broad O-H). ^1^H NMR (400 MHz, CDCl_3_) *δ* (ppm) 7.53–7.51 (m, 2H, Ph), 7.34–7.26 (m, 3H, Ph), 5.98–5.82 (m, 6H, 5x OCH_2_CH=CH_2_, H1 or H-1′), 5.70–5.61 (m, 1H, OCH_2_CH=CH_2_), 5.38–5.06 (m, 12H, 6xOCH_2_CH=CH_2)_, 5.02–4.96 (m, 3H, H1′, H7, 1 CH-O), 4.38–4.04 (m, 13H, 6 × OCH_2_CHCH_2_), 3.98 (dd, *J* = 11.4, 2.7 Hz 1H, H-6 or H-6′), 3.88 (dd, *J* = 11.7, 2.7 Hz, 1H, H-6 or H-6′), 3.78–3.47 (m, 8H, H6, H6′, 6H CH-O), 3.19 (dd, *J* = 9.7, 9.6 Hz, 1H, H-4 or H-4′), 3.10 (dd, *J* = 9.5, 9.6 Hz, 1H, H-4 or H-4′). ^13^C NMR (100 MHz, CDCl_3_) *δ* (ppm) 139.3 (Cq-Ar) 135.3, 135.2, 134.7, 134.5, 134.4, 134.3, (6x OCH_2_CH=CH_2_), 128.7 (2 × CHAr), 128.4, (CHAr), 128.3, (2 × CHAr), 118.0, 117.8, 117.7, 117.2, 116.9, 116.8 (6 × OCH_2_CH=CH_2_), 82.8 (C-1 or C-1′), 82.2, 82.1, 81.9, 79.05, 79.0 (5xCH), 78.3 (C-4 or C-4′), 77.7 (C4 or C4′), 74.5 (2 × OCH_2_CH=CH_2_), 74.4 (OCH_2_CH=CH_2_), 74.0 (OCH_2_CH=CH_2_), 72.6 (CH), 72.5 (CH), 71.3 (OCH_2_CH=CH_2_), 71.1 (OCH_2_CH=CH_2_), 63.2 (C-6 or C-6′), 62.2 (C-6 or C-6′), 46.1 (C-7). HRMS (ESI) *m*/*z* [M + Na]^+^ calculalted for [C_37_H_52_NaO_10_S_2_]^+^: 743.290, found 743.290.

### 3.3. Synthesis of Dithioketal-α,α-Diglycosides **8** and Characterization of Compound **3**

Diglycoside thioketal **8a.** In a tube, to a solution of cyclohexanone (1 equiv., 13.2 mg, 0.014 mL, 0.134 mmol) and anhydroglucose **5a** (2 equiv., 116 mg, 0.269 mmol) in CH_2_Cl_2_ (0.6 mL) was added (TMS)_2_S (5 equiv., 0.126 mL, 0.672 mmol) and TMSOTf (2 equiv., 0.0488 mL, 0.269 mmol). The tube was sealed and the mixture was heated at 60 °C for 18 h. The mixture was washed with saturated aqueous NaHCO_3_ (40 mL) and extracted with CH_2_Cl_2_ (3 × 50 mL). The organics layers were combined and washed with brine (40 mL), dried over Na_2_SO_4_ and concentrated under reduced pressure. The crude obtained was purified by flash column chromatography (Petroleum Ether/EtOAc, 8/2 to 1/1) to afford compound **8a** (61 mg) in 45% yield. ${\left[\alpha \right]}_{D}^{20}$ + 114 (*c* 1, CH_2_Cl_2_). IR 3463 (O-H, weak, broad), 2928 (C-H) cm^-1^. ^1^H NMR (400 MHz, CDCl_3_) *δ* (ppm) 7.31–7.13 (m, 30H, H-Ar), 5.79 (d, *J* = 5.4 Hz, 2H, H-1), 4.84–4.48 (m, 12H, Ph-CH_2_-O), 4.06 (dt, *J* = 10.1, 3.1 Hz, 2H, H-5), 3.73 (t, *J* = 9.5 Hz, 2H, H-3), 3.69 (m, 4H, H-6), 3.60 (dd, *J* = 9.8, 5.6 Hz, 2H, H-2), 3.46 (t, *J* = 9.3 Hz, 2H, H-4), 1.96 (t, *J* = 5.6 Hz, 4H, H-8), 1.54 (m, 4H, H-9), 1.31 (m, 2H, H-10). ^13^C NMR (100 MHz, CDCl_3_) *δ* (ppm) 138.5 (Cq-Ar,) 138.2 (Cq-Ar,), 138.1 (Cq-Ar) 128.64, 128.60, 128.54, 128.51, 128.18, 128.14, 128.09, 128.05, 127.84, 127.77, 127.67 (CH Ar), 83.4 (C-3), 82.6 (C-1), 79.6 (C-2), 77.4 (C-4), 75.7 (Ph-CH_2_-O), 75.3 (Ph-CH_2_-O), 73.0 (Ph-CH_2_-O), 72.7 (C-5), 64.1 (C-7), 62.0 (C-6), 39.9 (C-8), 25.3 (C-10), 23.1 (C-9). HRMS (ESI) *m*/*z* [M + Na]^+^ calculated for [C_60_H_68_O_10_S_2_Na]^+^: 1035.415; found 1035.416.

Diglycoside thioketal **8b.** In a tube, to a solution of anhydroglucose **5a** (2 equiv., 100 mg, 0.231 mmol) in CH_2_Cl_2_ (0.6 mL) was added (TMS)_2_S (2.8 equiv., 0.0608 mL, 0.324 mmol) and TMSOTf (2.2 equiv., 0.0461 mL, 0.254 mmol). The tube was sealed and the mixture was heated at 50 °C for 2 h. Then cyclobutanone (1 equiv., 8.1 mg, 0.00871 mL, 0.116 mmol) was added to the mixture at −70 °C and stirred 15 h. The mixture was washed with saturated aqueous NaHCO_3_ (40 mL) and extracted with CH_2_Cl_2_ (3 × 50 mL). The organics layers were combined and washed with brine (40 mL), dried over Na_2_SO_4_ and concentrated under reduced pressure. The crude obtained was purified by flash column chromatography (Petroleum Ether/EtOAc, 8/2 to 0/1) to afford compound **8b** (35 mg) in 31% yield. ${\left[\alpha \right]}_{D}^{20}$ + 87 (*c* 1, CH_2_Cl_2_). IR 3466 (O-H, weak, broad), 2925 (C-H) cm^−1^. ^1^H NMR (400 MHz, CDCl_3_) *δ* (ppm) 7.31–7.13 (m, 30H, H-Ar), 5.74 (d, *J* = 5.6 Hz, 2H, H-1), 4.79 (t, *J* = 10.9 Hz, 4H, O-CH_2_-Ph), 4.69 (d, *J* = 10.4 Hz, 2H, O-CH_2_-Ph) 4.57 (d, *J* = 10.9 Hz, 2H, O-CH_2_-Ph), 4.09 (dt, *J* = 9.7, 3.1 Hz, 2H, H-5), 3.79–3.66 (m, 6H, H-3, H-6), 3.60 (dd, *J* = 9.9, 5.7 Hz, 2H, H-2), 3.47 (dd, *J* = 9.6, 9.0 Hz, 2H, H-4), 2.58–2.47 (m, 2H, H-8a), 2.40–2.31 (m, 2H, H-8b), 2.06 (q, *J* = 7.6 Hz, 2H, H-9). ^13^C NMR (100 MHz, CDCl_3_) *δ* (ppm) 138.4, (Cq-Ar), 138.1 (Cq-Ar), 137.9 (Cq-Ar), 128.5, 128.4, 128.03, 127.97, 127.93, 127.7, 127.3 (CHAr), 83.8 (C-1), 83.2 (C-3), 79.4 (C-2), 77.3 (C-4), 75.6 (O-CH_2_-Ph), 75.1 (O-CH_2_-Ph), 72.7 (O-CH_2_-Ph), 72.5 (C-5), 61.8 (C-6), 59.1 (C-7), 38.9 (C-8), 18.0 (C-9). HRMS (ESI) *m*/*z* [M + Na]^+^ calculated for [C_58_H_64_O_10_S_2_ Na]^+^: 1007.383; found 1007.382.

Diglycoside thioketal **8c.** In a tube, to a solution of 3-pentanone (1 equiv., 9.96 mg, 0.0122 mL, 0.116 mmol) and anhydroglucose **5a** (2 equiv., 100 mg, 0.231 mmol) in CH_2_Cl_2_ (0.6 mL) was added (TMS)_2_S (5 equiv., 103 mg, 0.109 mL, 0.578 mmol) and TMSOTf (2 equiv., 51.4 mg, 0.0419 mL, 0.231 mmol). The tube was sealed and the mixture was heated at 50 °C for 2 h. The mixture was washed with saturated aqueous NaHCO_3_ (40 mL) and extracted with CH_2_Cl_2_ (3 × 50 mL). The organics layers were combined and washed with brine (40 mL), dried over Na_2_SO_4_ and concentrated under reduced pressure. The crude obtained was purified by flash column chromatography (Petroleum Ether/EtOAc, 8/2 to 4/6) to afford compound **8c** (13 mg) in 11% yield. ${\left[\alpha \right]}_{D}^{20}$ + 104 (*c* 1, CH_2_Cl_2_). IR 3465 (O-H, weak, broad), 2875 (C-H) cm^−1^. ^1^H NMR (400 MHz, CDCl_3_) *δ* (ppm) 7.40–7.15 (m, 30H, H-Ar), 5.83 (d, *J* = 5.7 Hz, 2H, H-1), 4.87 (d, *J* = 6.3 Hz, 2H, O-CH_2_-Ph), 4.85 (d, *J* = 6.2 Hz, 2H, O-CH_2_-Ph), 4.76 (d, *J* = 10.9 Hz, 2H, O-CH_2_-Ph), 4.66–4.56 (m, 6H, O-CH_2_-Ph), 4.10 (dt, *J* = 9.8, 3.2 Hz, 2H, H-5), 3.84–3.75 (m, 6H, H-3, H-6), 3.63 (dd, *J* = 9.9, 5.6 Hz, 2H, H-2), 3.52 (dd, *J* = 10.0, 8.9 Hz, 2H, H-4), 2.02–1.92 (m, 2H, H-8a), 1.92–1.81 (m, 2H, H-8b), 0.99 (t, *J* = 7.3 Hz, 6H, H-9). ^13^C NMR (100 MHz, CDCl_3_) *δ* (ppm) 138.5 (Cq-Ar), 138.2 (Cq-Ar), 138.1 (Cq-Ar) 128.64, 128.53, 128.50, 128.17, 128.06, 127.82, 127.76, 127.5 (CH Ar), 83.3 (C-3), 83.9 (C-1), 79.6 (C-2), 77.4 (C-4), 75.7 (Ph-CH_2_-O), 75.3, (Ph-CH_2_-O), 73.1 (Ph-CH_2_-O), 72.8 (C-5), 68.5 (C-7), 61.9 (C-6), 31.9 (C-8), 9.1 (C-9). HRMS (ESI) [M + K]^+^ calculated for [C_59_H_68_O_10_S_2_K]^+^: 1039.389; found 1039.394.

Diglycoside thioketal **3.** ${\left[\alpha \right]}_{D}^{20}$ + 128 (*c* 1, CH_2_Cl_2_). IR 2174 (C≡C, weak, sharp) cm^−1^. ^1^H NMR (400 MHz, CDCl_3_) *δ* (ppm) 5.56 (d, *J* = 5.7 Hz, 2H, H-1), 4.66–4.24 (m, 12H, H-9), 3.97 (dd, *J* = 9.9 and 5.7 Hz, 2H, H-2), 3.91–3.80 (m, 4H, H-5, H-6a), 3.73 (dd, *J* = 11.8, 5.2 Hz, 2H, H-6b), 3.63 (t, *J* = 9.2 Hz, 2H, H-3), 3.43 (t, *J* = 9.4 Hz, 2H, H-4), 1.68 (s, 6H, H-8), 1.12–0.98 (m, 126H, H-TIPS). ^13^C NMR (100 MHz, CDCl_3_) *δ* (ppm) 103.9, 103.7, 102.9 (3 × C-10), 88.9, 87.8, 87.5 (3 × C-11), 83.6 (C-1), 82.7 (C-3), 77.2 (C-2), 76.7 (C-4), 73.1 (C-5), 62.5 (C-6), 61.0, 60.7, 59.1 (3 × C-9), 58.7 (C-7), 32.9 (C-8), 18.7 (CH_3_-CH-Si), 11.3 (CH_3_-CH-Si). HRMS (ESI) *m*/*z* [M + Na]^+^ calculated for [C_87_H_160_O_10_S_2_Si_6_Na]^+^_:_ 1619.996; found 1619.989.

## 4. Conclusions

In conclusion, we have reported a one-pot, highly stereoselective access to dithioacetal-α,α-diglycosides, a class of disaccharide mimetics with almost no precedent. This highly convergent approach is based on the in situ formation of a thioglycoside intermediate via 1,6-anhydrosugars ring opening and its reaction with an aldehyde or a ketone. In this process, three C-S bounds are generated successively and two primary hydroxyl groups, that may be orthogonally protected or further functionalized, are released. The *pseudo* thiodisaccharides obtained could have interesting applications in glycobiology or asymmetric synthesis. Further applications of this approach to the synthesis of unprecedented disaccharide mimetics of biological interest are currently under investigation in our laboratory.

## Figures and Tables

**Table 1:**
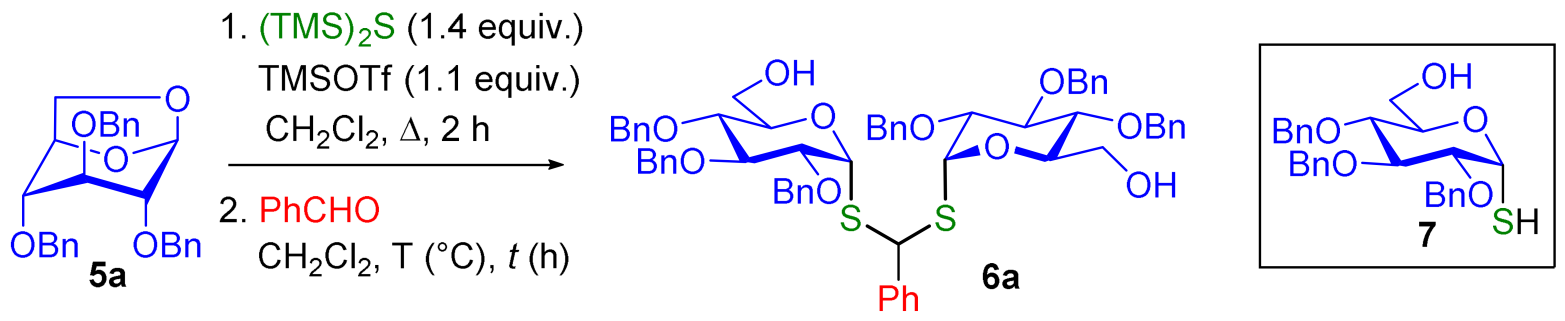
Optimization of the one-pot thioacetalization process.

Entry ^1^	PhCHO (equiv.)	T (°C) ^2^	*t* (h) ^3^	Yield (%) ^4^
1	0.5	25	20	13
2	1	−15	1.5	27
3	1	−30	1.5	45
4 ^5^	1	−30	1.5	60
5 ^5^	3	−78	1.5	45
6 ^5^	4	−78	2	42
7 ^5^	8	−78	1.5	41

^1^ The reactions are performed in sealed tubes. ^2^ T refers to the temperature of the second step. ^3^ *t* refers to the reaction time of the second step. ^4^ Isolated yields. ^5^ Reaction performed in the presence of 4Å molecular sieves.

**Table 2:**
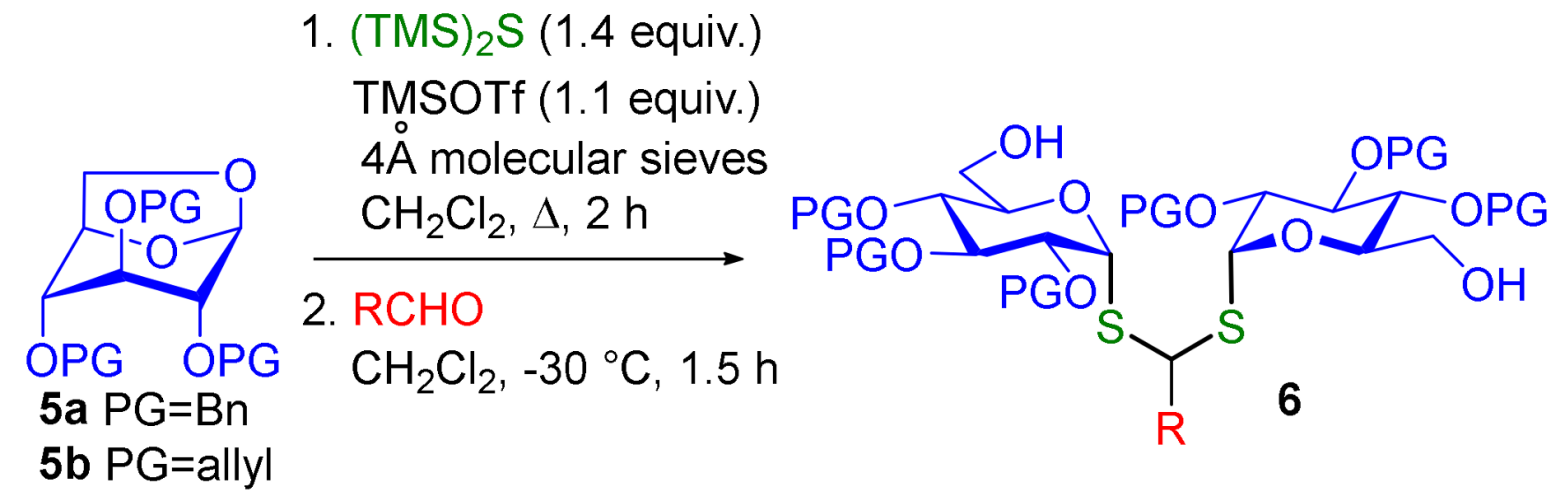
Scope of aldehydes in the one-pot formation of dithioacetal-α,α-diglycosides.

Entry	PG	RCHO	Product	Yield (%) ^1^
1	Bn	PhCHO	**6a**	60
2	Bn	4-CF_3_-PhCHO	**6b**	16
3	Bn	4-MeO-PhCHO	**6c**	45
4	Bn	*n*-C_3_H_7_CHO	**6d**	30
5	Bn	*n*-C_5_H_11_CHO	**6e**	33
6	Bn	*t*-BuCHO	**6f**	25
7	allyl	PhCHO	**6g**	50

^1^ Isolated yields.

## Supplementary Materials

Supplementary materials are available online.
