# Supplementary material for: One-Pot, Highly Stereoselective Synthesis of Dithioacetal-α,α-Diglycosides
Source: Molecules. 2018 Apr 15;23(4):914. doi: 10.3390/molecules23040914 (PMC6017313; doi:10.3390/molecules23040914)
Supplement: Supplementary file 1 [file molecules-23-00914-s001.pdf]

Supporting Information

One-pot, highly stereoselective synthesis of dithioacetal-α,α-diglycosides

Maria F. Céspedes Dávila[§], Jérémy P. Schneider[§], Amélie Godard, Damien Hazelard and Philippe Compain*

Laboratoire d'Innovation Moléculaire et Application (LIMA). Université de Strasbourg, Université de Haute-Alsace, CNRS (UMR 7042). Equipe Synthèse Organique et Molécules Bioactives (SYBIO), Ecole Européenne de Chimie, Polymères et Matériaux, 25 rue Becquerel, 67000 Strasbourg, France

E-Mail : philippe.compain@unistra.fr
[§]*Both authors contributed equally to the work*

Table of Contents:

General methods S2

Experimental procedures S2-S13

^{1}H and ^{13}C NMR of new compounds S14-S24

General Methods

Dichloromethane (CH_2Cl_2) was distilled over CaH_2 under Ar. Dried DMF was purchased from Sigma-Aldrich. Petroleum ether (b. p. 40-60 °C) was supplied in GPR Rectapur® quality. All reactions were performed in standard glassware under Ar unless otherwise specified. Flash chromatographies were performed on silica gel 60 (230-400 mesh, 0.040-0.063 mm) purchased from E. Merck. Thin Layer Chromatography (TLC) was performed on aluminum sheets coated with silica gel 60 F_{254} purchased from E. Merck. IR spectra (cm^{-1}) were recorded on a Perkin-Elmer Spectrum One spectrophotometer. NMR spectra were recorded on 300 MHz and 400 MHz spectrometers with solvent peaks as reference.[1] Carbon multiplicities were assigned by distortionless enhancement by polarization transfer (DEPT) experiments. The ^1H signals were assigned by 2D experiments (COSY, HSQC, HMBC). ESI-HRMS mass spectra were carried out on a Brucker MicroTOF spectrometer by the Analytical Service of the University of Strasbourg. Optical rotations were measured at 589 nm (sodium lamp) and 20 °C with an Anton Paar MCP 200 polarimeter with a path length of 1 dm.

Anhydroglucose 2

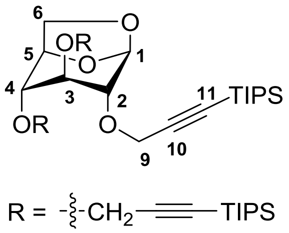

To a solution of 1,6-Anhydro-2,3,4-tri-O-propargyl-β-D-glucopyranose[1] (1 equiv., 675 mg, 2.44 mmol) in THF (60.9 mL) at -70 °C was added LiHMDS (4 equiv., 1 M, 9.77 mL, 9.77 mmol) dropwise. Then after 20 min TIPSCl (4 equiv., 1.94 g, 2.16 mL, 9.77 mmol) was added dropwise. The reaction mixture was stirred for 2h. The reaction was quenched with MeOH and the mixture was evaporated. Then saturated aqueous solution of NH_4Cl (40 mL) and Et_2O (40 mL) were added. The aqueous layer was extracted with Et_2O (2 x 40 mL). The combined organic extracts were washed with brine (40 mL), dried with Na_2SO_4, filtered and concentrated. The crude was purified by flash chromatography (Petroleum Ether/EtOAc 100/0 to 70/30), to afford **2** (1.15 g, 63%) as a yellow solid.

$[\boldsymbol{\alpha}]_{\boldsymbol{D}}^{\mathbf{20}}$ -30 (*c* 1, EtOH).

IR 2172 (C≡C, weak, sharp) cm^{-1}..

^{1}H NMR (400 MHz, $CDCl_3$) δ (ppm) 75.51 (s, 1H, H-1), 4,67 (d, J =5.0 Hz, 1H, H-5,) 4,47-4.25 (m, 6H, OCH_2C≡CTIPS), 3.97 (dd, J = 7.2 , 0.8 Hz, 1H, H-6), 3.83 (m, 1H, H-3), 3.74-3.67 (m, 2H, H-4, H-6), 3.60 (s, 1H, H-2), 1,01-1.10 (m, 63H, TIPS).

[1] Lepage, M. L.; Bodlenner, A.; Compain, P. *Eur. J. Org. Chem.* **2013**, 1963–1972.

^{13}C NMR (100 MHz, $CDCl_3$) δ (ppm) 103.0, 102.9, 102.8 (3xC-10), 100.4 (C-1), 88.8, 88.6 (3xC-11), 75.1, 74.7, 74.3, 74.1 (4xCH-O), 65.4 (C-6), 58.0, 57.8, 57.5 (3x$OCH_2C\equiv CTIPS$), 18.7 (CH_3-CH-Si), 11.3 (CH_3-CH-Si).

HRMS (ESI) m/z $[M+Na]^+$ calculated for $[C_{42}H_{76}O_5Si_3Na]^+$: 767.489; found 767.487.

Anhydroglucose 5a

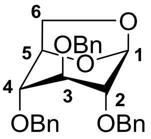

To a solution of levoglucosan (1 equiv, 5g, 30.5 mmol) in DMF (200 mL) at 0 ℃ was added benzyl bromide (3.5 equiv, 18.3 g, 12.8 mL, 106 mmol). Sodium hydride (60% in oil, 5 equiv, 6.11 g, 152 mmol) was added portionwise. The ice bath was removed, and the reaction was stirred overnight at r.t. Methanol (60 mL) was added, and 15 min later water was added (120 mL). The aqueous layer was extracted with EtOAc (3 × 200 mL), and the combined organic extracts were washed with brine (1 × 200 mL). The organic solution was dried over sodium sulfate, filtered, concentrated to an oil that was purified by flash chromatography (Petroleum Ether/EtOAc 8/2 to 6/4), to afford a solid that was then recrystallized from ethanol to afford **5a** (11.34 g, 26.23 mmol) as white crystals in 86% yield.

^{1}H NMR (300 MHz, $CDCl_3$) δ (ppm) 7.35-7.22 (m, 15H, Ph), 5.46 (s, 1H, H-1), 4,64-4,51 (m, 5H, H-5, O-CH_2-Ph), 4,45 (d, J = 12.1 Hz, 1H, O-CH_2-Ph) 4,41 (d, J = 12.1 Hz, 1H, O-CH_2-Ph), 3.91 (d, J = 7.2 Hz, 1H, H-6), 3.68 (t, J = 6.5 Hz, 1H, H-6), 3.59 (m, 1H, H-3), 3.35 (m, 2H, H-2, H-4).

MS (ESI): m/z $[M+K]^+$ calculated for $[C_{27}H_{28}O_5K]^+$: 471.16 found 471.16.

The analyses are in good agreement with the experimental data reported in literature.[2]

Anhydroglucose 5b

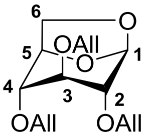

To a solution of levoglucosan (1 equiv, 1g, 6.11 mmol) in DMF (45 mL) at -20 ℃ was added sodium hydride (60% in oil, 4 equiv, 0.98 g, 24.4 mmol) portionwise. The cooling bath was allowed to reach 0 ℃ within 1h, then it was cooled again to -20 ℃ and allyl bromide (4.5 equiv, 2.4 mL, 27.6 mmol) was added under vigorous stirring. The reaction mixture was stirred overnight at rt. The mixture was diluted with water and extracted with diethyl ether (3

[2] Mcdevitt, J. P.; Lansbury, P. T. *J. Am. Chem. Soc.* **1996**, *118*, 3818–3828.

x 100 mL). The organic phases are combined and washed with water (5 x 80 mL), dried with Na_2SO_4, filtered, and concentrated to afford a yellow oil which was purified by flash chromatography (Petroleum Ether/EtOAc, 9/1 to 1/1), to give **5b** (1.63 g, 5.77 mmol) as a yellow oil in 95% yield.

^{1}H NMR (400 MHz, $CDCl_3$) δ (ppm) 5.92-5.79 (m, 3H, $OCH_2C\underline{H}CH_2$), 5.37 (s, 1H, H-1), 5.26-5.12 (m, 6H, $OCH_2CHC\underline{H_2}$), 4.51 (m, 1H, H-5), 4,08-4,02 (m, 6H, $OC\underline{H_2}CHCH_2$), 3.85 (dd, J = 7.1, 0.8 Hz, 1H, H-6), 3.64 (dd, J = 6.8, 6,0 Hz, 1H, H-6), 3.45 (m, 1H, H-3), 3.24 (m, , 1H, H-4), 3.21 (m, 1H, H-2)

The analyses are in good agreement with the experimental data reported in literature.[3]

General procedure for the synthesis of diglycoside thioketals 6

Molecular sieves 4Å (80 mg) were added to a tube. A solution of anhydroglucose **5** (1 equiv, 0.233 mmol) in CH_2Cl_2 (1 mL) was added via cannula to the flask. Bis (trimethylsilyl) sulfide (1.4 equiv, 58.3 mg, 0.0613 mL, 0.327 mmol) followed by trimethylsilyl trifluoromethane sulfonate (1.1 equiv, 57 mg, 0.0466 mL, 0.257 mmol) were added to the mixture. The tube was sealed and the medium was stirred at 60 °C. After 2h of reaction, a solution of the corresponding aldehyde (1 equiv, 0.233 mmol) in 0.3 mL of DCM was added at -30 °C. The reaction was stirred for 1h30. The mixture was warmed up to r.t. and washed with saturated aqueous $NaHCO_3$ (50 mL). The aqueous layer was extracted with CH_2Cl_2 (3x 50 mL). The organic layers were combined and washed with brine (50 mL), dried over sodium sulfate and concentrated under reduced pressure. The crude obtained was purified by flash column chromatography (Petroleum Ether/EtOAc) to afford compounds **6**.

Diglycoside thioketal 6a

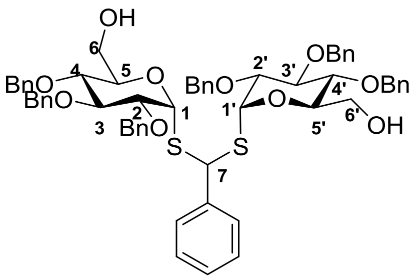

According to general procedure for the synthesis of diglycoside thioketals **6**, compound **6a** was obtained as a solid in 60% yield after flash column chromatography (Petroleum Ether/EtOAc, 8/2 to 4/6).

$[\alpha]_D^{20}$ +177 (c 0.7, $CHCl_3$)

IR 3431 cm^{-1} (broad O-H).

[3] Strašák,, T.; Karban,, J.; Šťastná, L. Č.; Maixnerová,, L.; Březinová, A.; Bernard, M.; Fajgar, R. *J. Organomet. Chem.* **2014**, *768,* 115-120.

^{1}H NMR (400 MHz, $CDCl_3$) δ (ppm) 7.44-7.22 (m, 35H, Ph), 5.95 (d, J = 5.5 Hz, 1H, H-1 or H-1'), 5.02 (s, 1H, H-7), 5.00-4.97 (m, 2H, O-C$\underline{H}_2$-Ph, H-1 or H-1'), 4.91-4.77 (m, 5H, O-C$\underline{H}_2$-Ph), 4.71 (d, J = 10.8 Hz, 1H, O-C$\underline{H}_2$-Ph), 4.62 (d, J = 11.4 Hz, 1H, O-C$\underline{H}_2$-Ph), 4.57 (d, J = 5.9 Hz, 1H, O-C$\underline{H}_2$-Ph), 4.54 (d, J = 5.7 Hz, 1H, O-C$\underline{H}_2$-Ph), 4.38 (d, J = 11.5 Hz, 1H, O-C$\underline{H}_2$-Ph), 4.32 (d, J = 11.5 Hz, 1H, O-C$\underline{H}_2$-Ph), 4.21 (m, 1H, H-5 or H-5'), 4.14 (m, 1H, H-5 or H-5'), 3.98-3.80 (m, 5H, H-2 or H-2', H-6, H-6', H3, H3'), 3.67 (dd, J = 9.5; 5.6 Hz, 1H, H-2 or H-2'), 3.63-3.55 (m, 2H, H-6, H6'), 3.37 (dd, J = 10.0; 8.8 Hz, 1H, H-4 or H-4'), 3.28 (dd, J = 9.9; 8.8 Hz, 1H, H-4 or H-4').

^{13}C NMR (100 MHz, $CDCl_3$) δ (ppm) 139.4 (Cq-Ar), 138.6 (Cq-Ar), 138.55 (Cq-Ar), 138.1 (Cq-Ar), 137.8 (Cq-Ar), 137.7 (Cq-Ar), 137.3 (Cq-Ar), 128.9-127.8 (35 x CHAr) 82.8 (CH) 82.7 (CH), 82.6 (CH), 81.9 (C-1 or C-1'), 79.4 (CH), 78.8 (CH), 77.8 (C-4 or C-4'), 78.4 (C-4 or C-4'), 75.9 (O-$\underline{C}H_2$-Ph), 75.6 (O-$\underline{C}H_2$-Ph), 75.2 (O-$\underline{C}H_2$-Ph), 72.8 (C5 or C-5'), 72.6 (C-5 or C5'), 72.3 (O-$\underline{C}H_2$-Ph), 71.9 (O-$\underline{C}H_2$-Ph), 63.0 (C-6 or C-6'), 62.2 (C-6 or C-6'), 46.2 (C-7).

HRMS (ESI) m/z $[M+K]^+$ calculated for $[C_{61}H_{64}O_{10}S_2K]^+$: 1059.357, found: 1059.366.

Diglycoside thioketal 6b

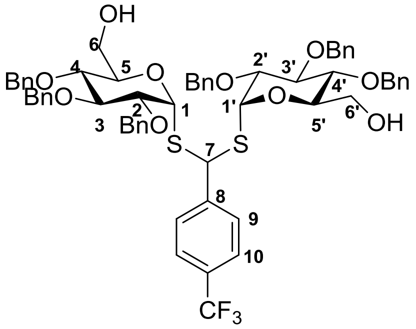

According to general procedure for the synthesis of diglycoside thioketals, **6** compound **6b** was obtained in 16% yield after flash column chromatography (Petroleum Ether/EtOAc, 8/2 to 0/1).

$[\boldsymbol{\alpha}]_{\boldsymbol{D}}^{\mathbf{20}}$ +152 (c 0.9, $CHCl_3$).

IR 3428 cm^{-1} (broad O-H).

^{1}H NMR (400 MHz, $CDCl_3$) δ (ppm) 7.69 (J = 8.2 Hz, 2H, H-10), 7.62 (J = 8.1 Hz, 2H, H-9), 7.45-7.09 (m, 30H, Ph), 5.93 (d, J = 5.5 Hz, 1H, H-1 or H-1'), 5.06 (s, 1H, H-7), 4.99 (d, J = 10.9 Hz, 1H, O-C$\underline{H}_2$-Ph), 4.91-4.78 (m, 6H, O-C$\underline{H}_2$-Ph, H-1 or H-1'), 4.71 (d, J = 10.9 Hz, 1H, O-C$\underline{H}_2$-Ph), 4.63 (d, J = 11.2 Hz, 1H, O-C$\underline{H}_2$-Ph), 4.55 (d, J = 10.5 Hz, 2H, O-C$\underline{H}_2$-Ph), 4.43 (d, J = 11.8 Hz, 1H, O-C$\underline{H}_2$-Ph), 4.39 (d, J = 11.7 Hz, 1H, O-C$\underline{H}_2$-Ph), 4.17 (ddd, J = 9.9, 7.4, 2.0 Hz, 1H, H-5 or H-5'), 4.09 (ddd, J = 9.7, 6.9, 2.8 Hz, 1H,H-5 or H-5'), 3.97-3.76 (m, 5H, H-6, H-6', H-3, H-3', H-2 or H-2') 3.68 (dd, J=9.4, 5.6 Hz, 1H, H-2 or H-2'), 3.64-3.54 (m, 2H, H-6, H-6') 3.35 (dd, J = 10.0; 8.9 Hz, 1H, H-4 or H-4'), 3.28 (dd, J = 10.0; 8.8 Hz, 1H, H-4 or H-4').

^{13}C NMR (100 MHz, CDCl$_3$) δ (ppm) 143.7 (C-8), 138.55 (Cq-Ar), 138.5 (Cq-Ar), 138.0 (Cq-Ar), 137.7 (Cq-Ar), 137.5 (Cq-Ar), 137.2 (Cq-Ar), 128.8-127.8 (34 x CHAr), 125.9 (C9, q, *J* C-F = 4 Hz), 82.65 (CH) 82.6 (CH), 82.55 (CH), 82.0 (CH, C-1 or C-1'), 79.4 (CH), 79.0 (C-2 or C-2'), 78.4 C-4 or C-4'), 77.7 (C-4 or C-4'), 75.9 (O-$\underline{C}H_2$-Ph), 75.6 (O-$\underline{C}H_2$-Ph), 75.2 (O-$\underline{C}H_2$-Ph), 72.85 (C-5 or C-5'), 72.8 (C-5 or C-5'), 72.5 (O-$\underline{C}H_2$-Ph), 72.4 (O-$\underline{C}H_2$-Ph), 63.1 (C-6 or C-6), 62.1 (C-6 or C-6'), 45.2 (C-7).

^{19}F NMR (376 MHz, CDCl$_3$) δ (ppm) -66.55 (3F, s, CF$_3$.)

HRMS (ESI) m/z [M+K]$^+$ calculated for $[C_{62}H_{63}F_1O_{10}S_2K]^+$: 1127.345, found: 1127.332.

Diglycoside thioketal 6c

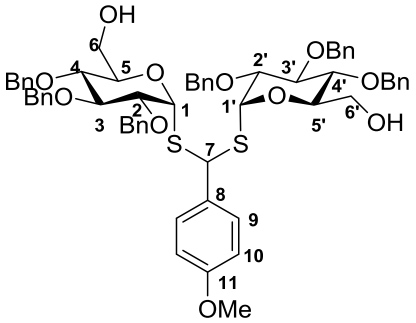

According to general procedure for the synthesis of diglycoside thioketals **6**, compound **6c** was obtained in 45% yield after flash column chromatography (Petroleum Ether/EtOAc, 8/2 to 0/1).

$[\alpha]_D^{20}$ +151(*c* 1.1, CHCl$_3$).

IR 3437 cm^{-1} (broad O-H).

^{1}H NMR (400 MHz, CDCl$_3$) δ (ppm) 7.49 (d, *J* = 8.8 Hz, 2H, H-10), 7.44-7.37 (m, 2H, Ph), 7.37-7.19 (m, 26H, Ph), 7.18-7.11 (m, 2H, Ph), 6.88 (*J* = 8.8 Hz, 2H, H-9), 5.93 (d, *J* = 5.4 Hz, 1H, H-1 or H-1'), 5.01-4.95 (m, 3H, H-7, H-1 or H-1', O-C$\underline{H}_2$-Ph), 4.90-4.63 (m, 3H, O-C$\underline{H}_2$-Ph), 4.81 (d, *J* = 5.0 Hz, 2H, O-C$\underline{H}_2$-Ph), 4.78 (d, *J* = 4.9 Hz, 1H, O-C$\underline{H}_2$-Ph), 4.69 (d, *J* = 10.8 Hz, 1H, O-C$\underline{H}_2$-Ph), 4.60 (d, *J* = 11.4 Hz, 1H, O-C$\underline{H}_2$-Ph), 4.55 (d, *J* = 5.4 Hz, 1H, O-C$\underline{H}_2$-Ph), 4.53 (d, *J* = 5.1 Hz, 1H, O-C$\underline{H}_2$-Ph), 4.39 (d, *J* = 11.5 Hz, 1H, O-C$\underline{H}_2$-Ph), 4.32 (d, *J* = 11.6 Hz, 1H, O-C$\underline{H}_2$-Ph), 4.20 (ddd, *J* = 10.1, 7.4, 2.5 Hz, 1H, H-5 or H-5'), 4.13 (ddd, *J* = 9.7, 6.9, 2.8 Hz, 1H, H-5 or H-5'), 3.96-3.79 (m, 5H, H-6, H-6', H-3, H-3', H-2 or H-2'), 3.80 (s, 3H, OC$\underline{H}_3$), 3.67 (dd, *J* = 9.5; 5.6 Hz, 1H, H-2 or H-2'), 3.64-3.54 (m, 2H, H-6, H-6') 3.35 (dd, *J* = 9.8; 8.9 Hz, 1H, H-4 or H-4'), 3.27 (dd, *J* = 10.0; 8.9 Hz, 1H, H-4 or H-4').

^{13}C NMR (100 MHz, CDCl$_3$) δ (ppm) 159.7 (C-11), 138.65 (Cq-Ar), 138.6 (Cq-Ar), 138.1 (Cq-Ar), 137.8 (Cq-Ar), 137.7 (Cq-Ar), 137.4 (Cq-Ar), 131.3 (C-8), 129.5 (C10), 128.7-127.7 (34 x CHAr), 114.2 (C-9), 82.75 (CH) 82.7 (CH), 82.6 (CH), 81.9 (C-1 or C-1'), 79.4 (CH), 78.9 (C-2 or C-2'), 78.4 (C-4 or C-4'), 77.8 (C-4 or C-4'), 75.9 (O-$\underline{C}H_2$-Ph), 75.6 (O-$\underline{C}H_2$-

Ph), 75.1 (O-CH2-Ph), 72.7 (C-5 or C-5'), 72.5 (C-5 or C-5'), 72.2 (O-CH2-Ph), 71.9 (O-CH2-Ph), 63.0 (C-6 or C-6'), 62.1 (C-6 or C-6'), 55.5 (OCH_3), 45.7 (C-7).

HRMS (ESI) m/z $[M+K]^+$ calculated for $[C_{62}H_{66}O_{11}S_2K]^+$: 1089.368, found: 1089.367.

Diglycoside thioketal 6d

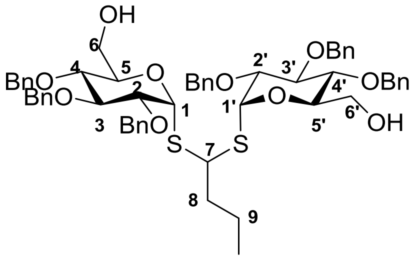

According to general procedure for the synthesis of diglycoside thioketals **6**, compound **6d** was obtained in 30% yield after flash column chromatography (Petroleum Ether/EtOAc, 8/2 to 4/6).

$[\boldsymbol{\alpha}]_{\boldsymbol{D}}^{\mathbf{20}}$ +136 (*c* 1, $CHCl_3$).

IR 3440 cm^{-1} (broad O-H).

^{1}H NMR (400 MHz, $CDCl_3$) δ (ppm) 7.35-7.26 (m, 30H, Ph), 5.85 (d, *J* = 4.5 Hz, 1H, H-1 or H-1'), 5.37 (d, *J* = 5.4 Hz, 1H, H-1 or H-1'), 5.01-4.95 (m, 2H, O-CH2-Ph), 4.93 (s, 1H, O-CH2-Ph), 4.90-4.85 (m, 2H, O-CH2-Ph), 4.84 (s, 1H, O-CH2-Ph), 4.79 (t, *J* = 11.1 Hz, 2H, O-CH2-Ph), 4.74 (m, 1H, O-CH2-Ph), 4.71-4.70 (m, 2H, O-CH2-Ph), 4.63 -4.55 (m, 5H, O-CH2-Ph), 4.16-4.09 (m, 1H, H5 or H-5'), 4.05-3.95 (m, 2H, H-7, H-4 or H5'), 3.91-3.75 (m, 6H, H-6, H-6', H-3, H-3', H-2, H2'), 3.68-3.5 (m, 2H, H-6, H6'), 3.47-3.41 (m, 1H, H-4 or H-4'), 3.36 (dd, *J* = 10.1; 8.4 Hz, 1H, H-4 or H-4'), 1.93-1.87 (m, 2H, H-8), 1.76-1.64 (m, 1H, H-9), 1.50-1.39 (m, 1H, H-9), 0.96-0.92 (m, 3H, H-10).

^{13}C NMR (100 MHz, $CDCl_3$) δ (ppm) 138.8 (Cq-Ar), 138.6 (Cq-Ar), 138.1 (Cq-Ar), 137.8 (Cq-Ar), 137.7 (Cq-Ar), 137.65 (Cq-Ar),128.7-128.0 (30 x CHAr), 82.8 (CH), 82.52 (CH), 82.5 (CH), 81.3 (C-1 or C-1'), 79.6 (CH), 79.3 (CH), 78.4 (C-4 or C4'), 77.4 (C4 or C-4'), 75.9 (CO-CH2-Ph), 75.8 (O-CH2-Ph), 75.6 (O-CH2-Ph), 75.4 (O-CH2-Ph), 72.9 (C5 or C5'), 72.5 (O-CH2-Ph), 72.3 (C5 or C5'), 72.2 (O-CH2-Ph), 62.8 (C-6 or C6'), 61.9 (C6 or C-6'), 45.0 (C-7), 38.5 (C-8), 20.5 (C-9), 13.9 (C-10).

HRMS (ESI) m/z $[M+K]^+$ calculated for $[C_{59}H_{68}O_{10}S_2Na]^+$: 1023.415, found: 1023.420.

Diglycoside thioketal 6e

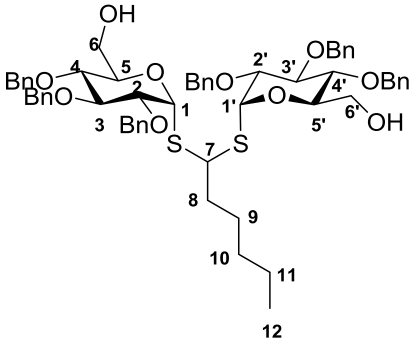

According to general procedure for the synthesis of diglycoside thioketals **6**, compound **6e** was obtained in 33% yield after flash column chromatography (Petroleum Ether/EtOAc, 8/2 to 0/1).

$[\boldsymbol{\alpha}]_{\boldsymbol{D}}^{\mathbf{20}}$ +173 (*c* 0.95, $CHCl_3$).

IR 3452 cm^{-1} (broad O-H).

^{1}H NMR (400 MHz, $CDCl_3$) δ (ppm) 7.45-7.17 (m, 30H, Ph), 5.85 (d, *J* = 4.3 Hz, 1H, H-1 or H-1'), 5.38 (d, *J* = 5.3 Hz, 1H, H-1 or H-1'), 5.02-4.54 (m, 12H, O-C<u>H</u>$_2$-Ph), 4.11 (ddd, *J* = 10.1, 6.8, 2.5 Hz, 1H, H-5 or H-5'), 4.05-3.93 (m, 2H, H-5 or H-5', H-7), 3.91-3.75 (m, 6H, H-6, H-6', H-3, H-3', H-2, H2'), 3.66 (dd, *J* = 12.6; 5.8 Hz, 1H, H-6 or H-6'),), 3.59 (dd, *J* = 11.6, 6.6 Hz, 1H, H-6 or H-6'), 3.48-3.42 (m, 1H, H-4 or H-4'), 3.36 (dd, *J* = 9.9; 8.6 Hz, 1H, H-4 or H-4'), 1.96-1.88 (m, 2H, H-8), 1.76-1.61 (m, 1H, H-9), 1.46-1.36 (m, 1H, H-9), 1.36-1.25 (m, 4H, H-10, H11), 0.97-0.85 (m, 3H, H-12).

^{13}C NMR (100 MHz, $CDCl_3$) δ (ppm) 138.8 (Cq-Ar), 138.6 (Cq-Ar), 138.1 (Cq-Ar), 137.8 (Cq-Ar), 137.7 (Cq-Ar), 137.6 (Cq-Ar), 128.7-127.7 (30 x CHAr), 82.8 (CH), 82.55 (CH), 82.5 (CH), 81.4 (C-1 or C-1'), 79.5 (CH), 79.3 (CH), 78.4 (C4 or C-4'), 77.4 (C-4 or C4'), 75.9 (O-<u>C</u>H$_2$-Ph), 75.8 (O-<u>C</u>H$_2$-Ph), 75.6 (O-<u>C</u>H$_2$-Ph), 75.4 (O-<u>C</u>H$_2$-Ph), 72.8 (O-<u>C</u>H$_2$-Ph), 72.5 (C-5 or C-5'), 72.3 (C-5 or C-5'), 72.25 (O-<u>C</u>H$_2$-Ph), 62.8 (C-6 or C-6'), 61.9 (C-6 or C-6'), 45.4 (C-7), 36.3 (C-8), 31.7 (C-10 or C-11), 26.7 (C-9), 22.7 (C-10 or C-11), 14.2 (C-12).

HRMS (ESI) m/z $[M+K]^+$ calculated for $[C_{60}H_{70}O_{10}S_2K]^+$: 1053.404, , found: 1053.391.

Diglycoside thioketal 6f

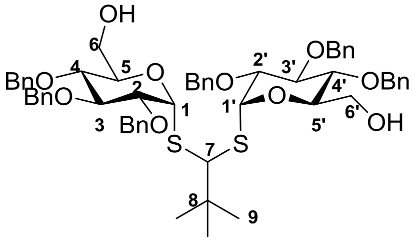

According to general procedure for the synthesis of diglycoside thioketals **6**, compound **6f** was obtained as a solid in 25% yield after flash column chromatography (Petroleum Ether/EtOAc, 8/2 to 4/6).

$[\boldsymbol{\alpha}]_{\boldsymbol{D}}^{\mathbf{20}}$ +128 (*c* 1.3, $CHCl_3$).

IR 3445 cm^{-1} (broad O-H).

^{1}H NMR (400 MHz, $CDCl_3$) δ (ppm) 7.39-7.29 (m, 30H, Ph), 5.76 (d, *J* = 4.8 Hz, 1H, H-1 or H-1'), 5.38 (d, *J* = 5.4 Hz, 1H, H-1 or H-1'), 5.01-4.66 (m, 9H, O-C$\underline{H}_2$-Ph), 4.61 (m, 2H, O-C$\underline{H}_2$-Ph), 4.63-456 (m, 1H, O-C$\underline{H}_2$-Ph), 4.10-4.05 (m, 1H, H-5 or H-5'), 4.03-3.98 (m, 1H, H-5 or H-5'), 3.90-3.77 (m, 6H, H-7, H-3, H-3', H6, H6', H-2 or H-2'), 3.76 (dd, *J* = 9.5; 5.4 Hz, 1H, H-2 or H-2'), 3.66 (dd, *J* = 12.3; 5.9 Hz, 1H, H-6 or H-6'), 3.59 (dd, *J* = 11.6, 6.2 Hz, 1H, H-6 or H-6'), 3.44 (t, *J* = 9.3 Hz, 1H, H-4 or H-4'), 3.36 (t, *J* = 9.2 Hz, 1H, H-4 or H-4'), 1.19 (s, 9H, H-9).

^{13}C NMR (100 MHz, $CDCl_3$) δ (ppm) 138.9 (Cq-Ar), 138.5 (Cq-Ar), 138.0 (Cq-Ar), 137.9 (Cq-Ar), 137.6 (Cq-Ar), 137.5 (Cq-Ar), 128.7-127.7, (30xCHAr), 86.0 (CH, C-1'), 83.0 (CH), 82.0 (CH), 81.3 (CH, C-1), 79.8 (C-2'), 79.0 (CH), 78.4 (C-4 or C-4'), 77.7 (C-4 or C-4') 75.8 (CH_2, O-$\underline{C}H_2$-Ph), 75.6 (CH2, O-$\underline{C}H_2$-Ph), 75.4 (O-$\underline{C}H_2$-Ph), 73.8 (O-$\underline{C}H_2$-Ph), 73.0 (C-5 or C-5'), 72.2 (CH2, O-$\underline{C}H_2$-Ph), 72.1 (C-5 or C-5') 62.8 (C-6 or C-6'), 62.2 (C-6 or C-6'), 59.6 (CH, C-7), 38.6 (C, C-8), 28.3 (3x CH_3, C-9.)

HRMS (ESI) m/z $[M+Na]^+$ calculated for $[C_{58}H_{66}O_{10}S_2Na]^+$: 1009.399, found: 1009.404.

Diglycoside thioketal 6g

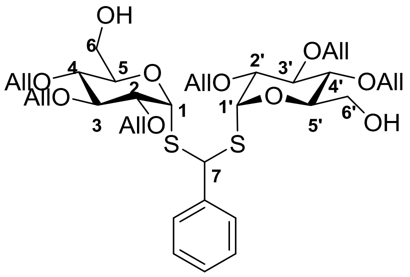

According to general procedure for the synthesis of diglycoside thioketals **6**, compound **6g** was obtained in 50% yield after flash column chromatography (Petroleum Ether/EtOAc, 9/1 to 1/9)

$[\boldsymbol{\alpha}]_{\boldsymbol{D}}^{\mathbf{20}}$ +388 (c 0.51, $CHCl_3$).

IR 3425 cm^{-1} (broad O-H).

^{1}H NMR (400 MHz, $CDCl_3$) δ (ppm) 7.53-7.51 (m, 2H, Ph), 7.34-7.26 (m, 3H, Ph), 5.98-5.82 (m, 6H, 5x $OCH_2C\underline{H}=CH_2$, H1 or H-1'), 5.70-5.61 (m, 1H, $OCH_2C\underline{H}=CH_2$), 5.38-5.06 (m, 12H, 6x$OCH_2CH=C\underline{H_2}$), 5.02-4.96 (m, 3H, H1', H7, 1 CH-O), 4.38-4.04 (m, 13H, 6x $OC\underline{H_2}CHCH_2$), 3.98 (dd, J = 11.4, 2.7 Hz 1H, H-6 or H-6'), 3.88 (dd, J = 11.7, 2.7 Hz, 1H, H-6 or H-6'), 3.78-3.47 (m, 8H, H6, H6', 6H CH-O), 3.19 (dd, J = 9.7, 9.6 Hz, 1H, H-4 or H-4'), 3.10 (dd, J = 9.5, 9.6 Hz, 1H, H-4 or H-4').

^{13}C NMR (100 MHz, $CDCl_3$) δ (ppm) 139.3 (CqAr) 135.3, 135.2, 134.7, 134.5, 134.4, 134.3, (6x $OCH_2\underline{C}H=CH_2$), 128.7 (2xCH_{Ar}), 128.4, (CHAr), 128.3, (2xCHAr), 118.0, 117.8, 117.7, 117.2, 116.9, 116.8 (6x $OCH_2CH=\underline{C}H_2$), 82.8 (C-1 or C-1'), 82.2, 82.1, 81.9, 79.05, 79.0 (5xCH), 78.3 (C-4 or C-4'), 77.7 (C4 or C4'), 74.5 (2x $O\underline{C}H_2CH=CH_2$), 74.4 ($O\underline{C}H_2CH=CH_2$), 74.0 ($O\underline{C}H_2CH=CH_2$), 72.6 (CH), 72.5 (CH), 71.3 ($O\underline{C}H_2CH=CH_2$), 71.1 ($O\underline{C}H_2CH=CH_2$), 63.2 (C-6 or C-6'), 62.2 (C-6 or C-6'), 46.1 (C-7).

HRMS (ESI) m/z $[M+Na]^+$ calculalted for $[C_{37}H_{52}O_{10}S_2Na]^+$: 743.290, found 743.290.

Diglycoside thioketal 8a

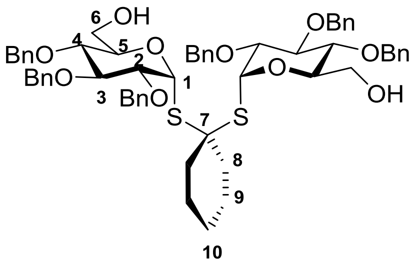

In a tube, to a solution of cyclohexanone (1 equiv, 13.2 mg, 0.014 mL, 0.134 mmol) and anhydroglucose **5a** (2 equiv, 116 mg, 0.269 mmol) in CH_2Cl_2 (0.6 mL) was added $(TMS)_2S$ (5 equiv, 0.126 mL, 0.672 mmol) and TMSOTf (2 eq., 0.0488 mL, 0.269 mmol). The tube

was sealed and the mixture was heated at 60 ℃ C for 18h. The mixture was washed with saturated aqueous $NaHCO_3$ (40 mL) and extracted with CH_2Cl_2 (3x 50 mL). The organics layers were combined and washed with brine (40 mL), dried over Na_2SO_4 and concentrated under reduced pressure. The crude obtained was purified by flash column chromatography (Petroleum Ether/EtOAc, 8/2 to 1/1) to afford compound **8a** (61 mg) in 45% yield.

$[\alpha]_D^{20}$ +114 (*c* 1, CH_2Cl_2).

IR 3463 (O-H, weak, broad), 2928 (C-H) cm^{-1}

^{1}H NMR (400 MHz, $CDCl_3$) δ (ppm) 7.31-7.13 (m, 30H, H-Ar), 5.79 (d, *J* = 5.4 Hz, 2H, H-1), 4.84-4.48 (m, 12H, Ph-C<u>H</u>2-O), 4.06 (dt, *J* = 10.1, 3.1 Hz, 2H, H-5), 3.73 (t, *J* = 9.5 Hz, 2H, H-3), 3.69 (m, 4H, H-6), 3.60 (dd, *J* = 9.8, 5.6 Hz, 2H, H-2), 3.46 (t, *J* = 9.3 Hz, 2H, H-4), 1.96 (t, *J* = 5.6 Hz, 4H, H-8), 1.54 (m, 4H, H-9), 1.31 (m, 2H, H-10).

^{13}C NMR (100 MHz, $CDCl_3$) δ (ppm) 138.5 (Cq-Ar,) 138.2 (Cq-Ar,), 138.1 (Cq-Ar,) 128.64, 128.60, 128.54, 128.51, 128.18, 128.14, 128.09, 128.05, 127.84, 127.77, 127.67 (<u>C</u>H Ar), 83.4 (C-3), 82.6 (C-1), 79.6 (C-2), 77.4 (C-4), 75.7 (Ph-<u>C</u>H_2-O), 75.3(Ph-<u>C</u>H_2-O), 73.0 (Ph-<u>C</u>H_2-O), 72.7 (C-5), 64.1 (C-7), 62.0 (C-6), 39.9 (C-8), 25.3 (C-10), 23.1 (C-9).

HRMS (ESI) m/z $[M + Na]^+$ calculated for $[C_{60}H_{68}O_{10}S_2Na]^+$: 1035.415; found 1035.416.

Diglycoside thioketal 8b

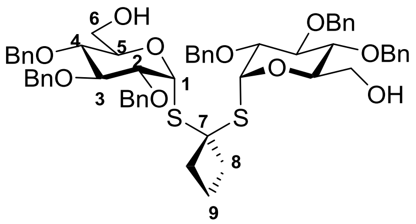

In a tube, to a solution of anhydroglucose **5a** (2 equiv, 100 mg, 0.231 mmol) in CH_2Cl_2 (0.6 mL) was added $(TMS)_2S$ (2.8 eq., 0.0608 mL, 0.324 mmol) and TMSOTf (2.2 eq., 0.0461 mL, 0.254 mmol). The tube was sealed and the mixture was heated at 50 ℃ for 2h. Then cyclobutanone (1 equiv, 8.1 mg, 0.00871 mL, 0.116 mmol) was added to the mixture at -70 ℃ and stirred 15h. The mixture was washed with saturated aqueous $NaHCO_3$ (40 mL) and extracted with CH_2Cl_2 (3x 50 mL). The organics layers were combined and washed with brine (40 mL), dried over Na_2SO_4 and concentrated under reduced pressure. The crude obtained was purified by flash column chromatography (Petroleum Ether/EtOAc, 8/2 to 0/1) to afford compound **8b** (35 mg) in 31% yield.

$[\alpha]_D^{20}$ +87 (*c* 1, CH_2Cl_2).

IR 3466 (O-H, weak, broad), 2925 (C-H) cm^{-1}

^{1}H NMR (400 MHz, $CDCl_3$) δ (ppm) 7.31-7.13 (m, 30H, H-Ar), 5.74 (d, *J* = 5.6 Hz, 2H, H-1), 4.79 (t, *J* = 10.9 Hz, 4H, O-CH_2-Ph), 4.69 (d, *J* = 10.4 Hz, 2H, O-CH_2-Ph) 4.57 (d, *J* =

10.9 Hz, 2H, O-CH_2-Ph), 4.09 (dt, *J* = 9.7, 3.1 Hz, 2H, H-5), 3.79-3.66 (m, 6H, H-3, H-6), 3.60 (dd, *J* = 9.9, 5.7 Hz, 2H, H-2), 3.47 (dd, *J* = 9.6, 9.0 Hz, 2H, H-4), 2.58-2.47 (m, 2H, H-8a), 2.40-2.31 (m, 2H, H-8b), 2.06 (q, *J* = 7.6 Hz, 2H, H-9).

^{13}C NMR (100 MHz, $CDCl_3$) δ (ppm) 138.4, (Cq-Ar), 138.1 (Cq-Ar), 137.9 (Cq-Ar), 128.5, 128.4, 128.03, 127.97, 127.93, 127.7, 127.3 (CHAr), 83.8 (C-1), 83.2 (C-3), 79.4 (C-2), 77.3 (C-4), 75.6 (O-CH_2-Ph), 75.1 (O-CH_2-Ph), 72.7 (O-CH_2-Ph), 72.5 (C-5), 61.8 (C-6), 59.1 (C-7), 38.9 (C-8), 18.0 (C-9).

HRMS (ESI) m/z $[M+Na]^+$ calculated for $[C_{58}H_{64}O_{10}S_2\ Na]^+$: 1007.383; found 1007.382.

Diglycoside thioketal 8c

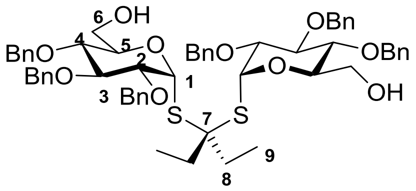

In a tube, to a solution of 3-pentanone (1 equiv, 9.96 mg, 0.0122 mL, 0.116 mmol) and anhydroglucose **5a** (2 equiv, 100 mg, 0.231 mmol) in CH_2Cl_2 (0.6 mL) was added $(TMS)_2S$ (5 equiv, 103 mg, 0.109 mL, 0.578 mmol) and TMSOTf (2 equiv, 51.4 mg, 0.0419 mL, 0.231 mmol). The tube was sealed and the mixture was heated at 50 ℃ for 2h. The mixture was washed with saturated aqueous $NaHCO_3$ (40 mL) and extracted with CH_2Cl_2 (3x 50 mL). The organics layers were combined and washed with brine (40 mL), dried over Na_2SO_4 and concentrated under reduced pressure. The crude obtained was purified by flash column chromatography (Petroleum Ether/EtOAc, 8/2 to 4/6) to afford compound **8c** (13 mg) in 11% yield.

$[\boldsymbol{\alpha}]_{\boldsymbol{D}}^{\mathbf{20}}$ +104 (*c* 1, CH_2Cl_2).

IR 3465 (O-H, weak, broad), 2875 (C-H) cm^{-1}

^{1}H NMR (400 MHz, $CDCl_3$) δ (ppm) 7.40-7.15 (m, 30H, H-Ar), 5.83 (d, *J* = 5.7 Hz, 2H, H-1), 4.87 (d, *J* = 6.3 Hz, 2H, O-CH_2-Ph), 4.85 (d, *J* = 6.2 Hz, 2H, O-CH_2-Ph), 4.76 (d, *J* = 10.9 Hz, 2H, O-CH_2-Ph), 4.66-4.56 (m, 6H, O-CH_2-Ph), 4.10 (dt, *J* = 9.8, 3.2 Hz, 2H, H-5), 3.84-3.75 (m, 6H, H-3, H-6), 3.63 (dd, *J* = 9.9, 5.6 Hz, 2H, H-2), 3.52 (dd, *J* = 10.0, 8.9 Hz, 2H, H-4), 2.02-1.92 (m, 2H, H-8a), 1.92-1.81 (m, 2H, H-8b), 0.99 (t, *J* = 7.3 Hz, 6H, H-9).

^{13}C NMR (100 MHz, $CDCl_3$) δ (ppm) 138.5 (Cq-Ar), 138.2 (Cq-Ar), 138.1 (Cq-Ar) 128.64, 128.53, 128.50, 128.17, 128.06, 127.82, 127.76, 127.5 (CH Ar), 83.3 (C-3), 83.9 (C-1), 79.6 (C-2), 77.4 (C-4), 75.7 (Ph-CH_2-O), 75.3, (Ph-CH_2-O), 73.1 (Ph-CH_2-O), 72.8 (C-5), 68.5 (C-7), 61.9 (C-6), 31.9 (C-8), 9.1 (C-9).

HRMS (ESI) m/z $[M+K]^+$ calculated for $[C_{59}H_{68}O_{10}S_2K]^+$: 1039.389; found 1039.394.

Data of diglycoside thioketal 3

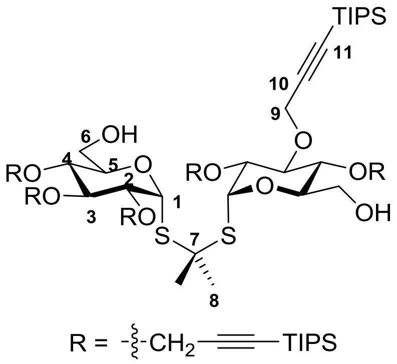

$[\alpha]_D^{20}$ +128 (*c* 1, CH_2Cl_2).

IR 2174 (C≡C, weak, sharp) cm^{-1}

^{1}H NMR (400 MHz, $CDCl_3$) δ (ppm) 5.56 (d, *J* = 5.7 Hz, 2H, H-1), 4.66-4.24 (m, 12H, H-9), 3.97 (dd, *J* = 9.9 and 5.7 Hz, 2H, H-2), 3.91-3.80 (m, 4H, H-5, H-6a), 3.73 (dd, *J* = 11.8, 5.2 Hz, 2H, H-6b), 3.63 (t, *J* = 9.2 Hz, 2H, H-3), 3.43 (t, *J* = 9.4 Hz, 2H, H-4), 1.68 (s, 6H, H-8), 1.12-0.98 (m, 126H, H-TIPS).

^{13}C NMR (100 MHz, $CDCl_3$) δ (ppm) 103.9, 103.7, 102.9 (3xC-10), 88.9, 87.8, 87.5 (3xC-11), 83.6 (C-1), 82.7 (C-3), 77.2 (C-2), 76.7 (C-4), 73.1 (C-5), 62.5 (C-6), 61.0, 60.7, 59.1 (3xC-9), 58.7 (C-7), 32.9 (C-8), 18.7 ($\underline{C}H_3$-CH-Si), 11.3 (CH_3-$\underline{C}$H-Si).

HRMS (ESI) m/z $[M+Na]^+$ calculated for $[C_{87}H_{160}O_{10}S_2Si_6Na]^+$: 1619.996; found 1619.989.

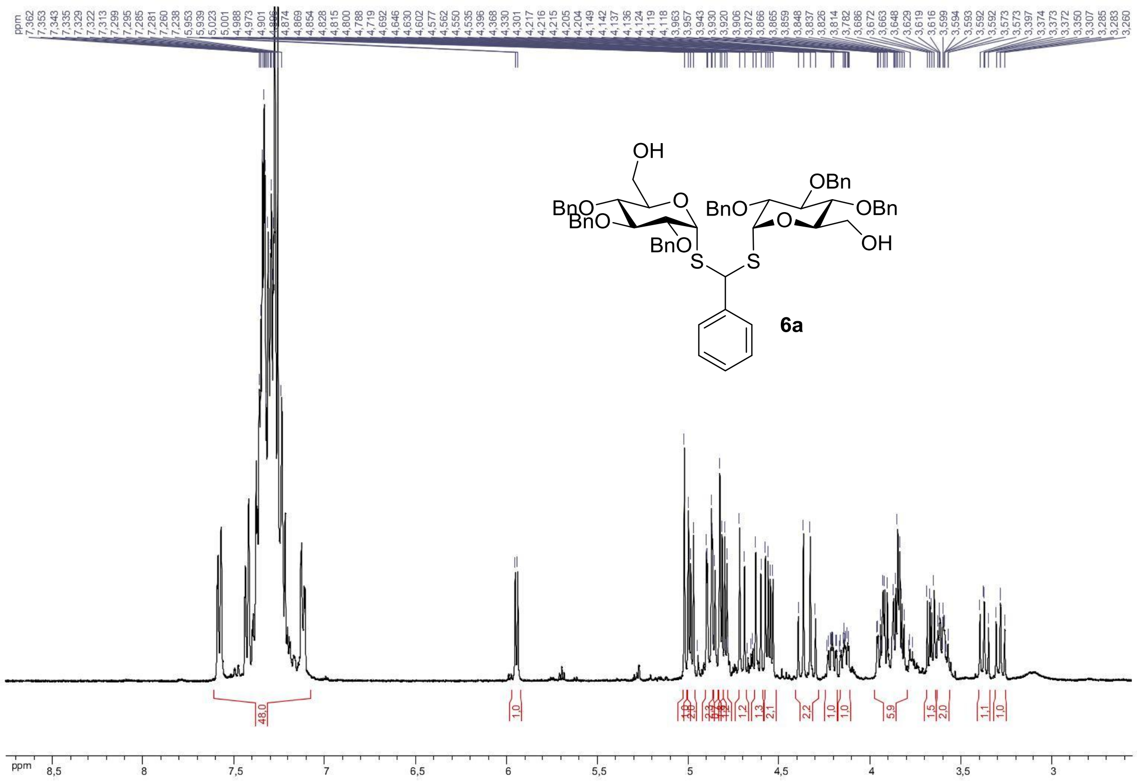

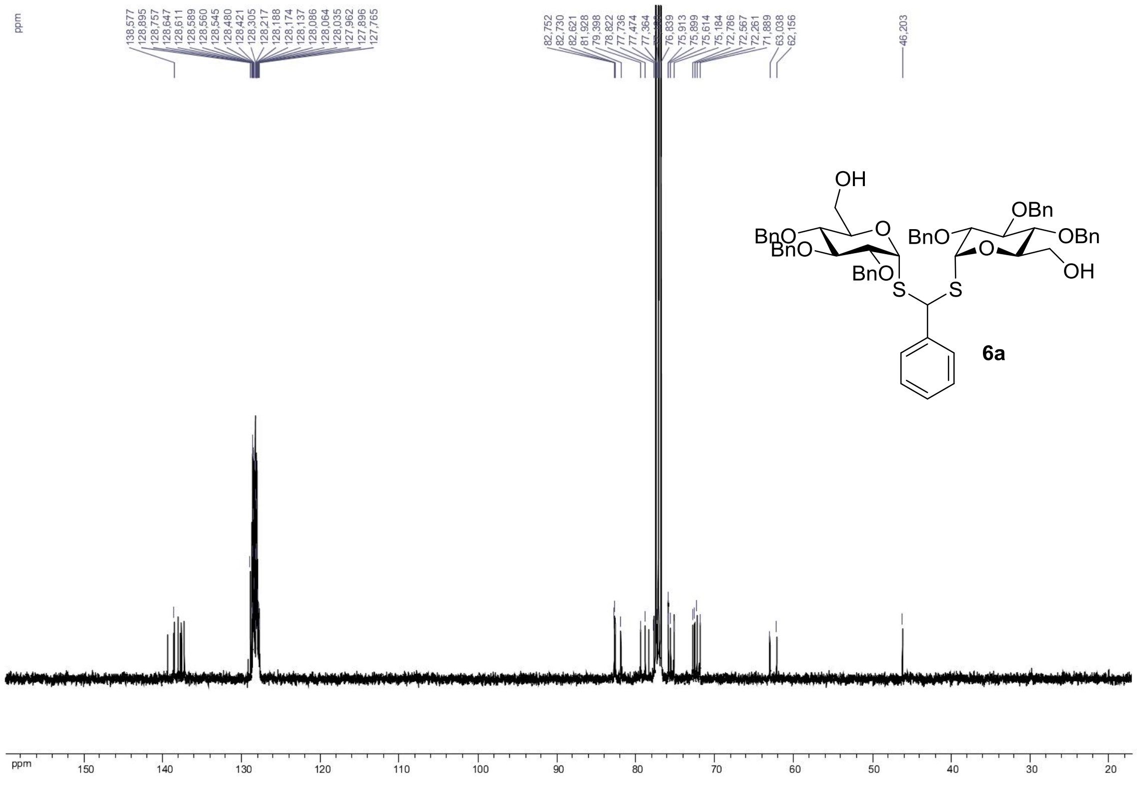

Fig 1. ^{1}H NMR (400 MHz, $CDCl_3$) and ^{13}C (100 MHz, $CDCl_3$) spectra of compound **6a**.

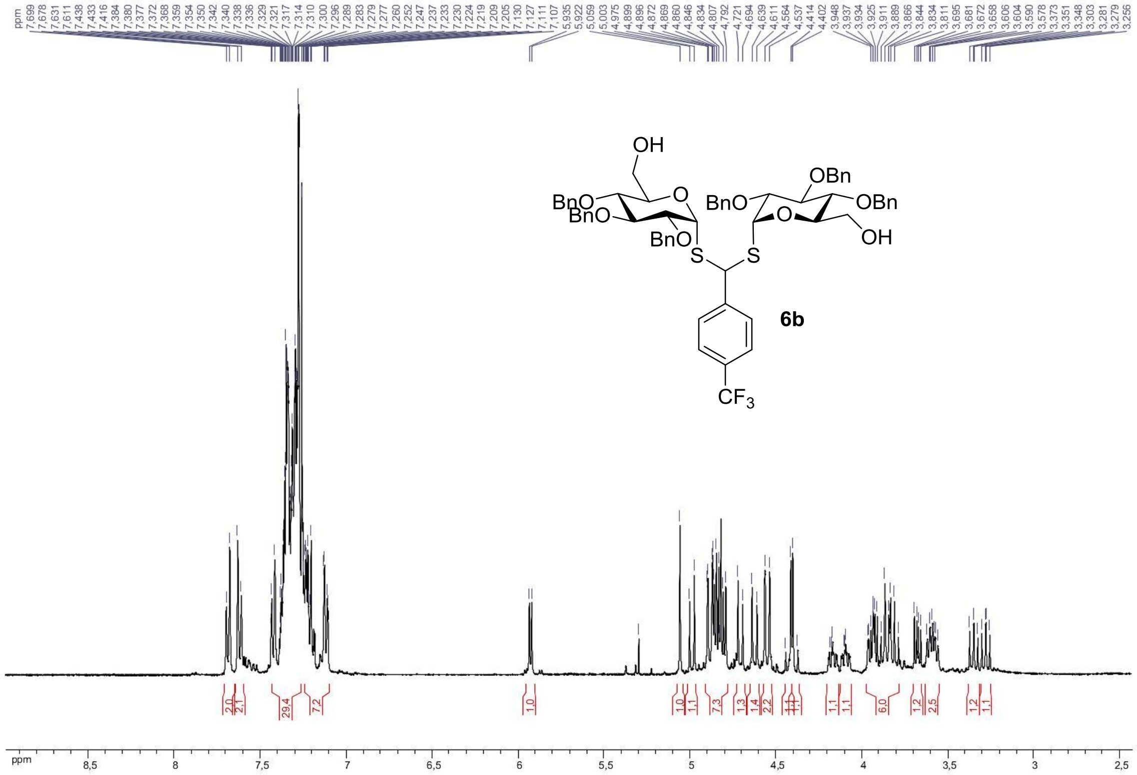

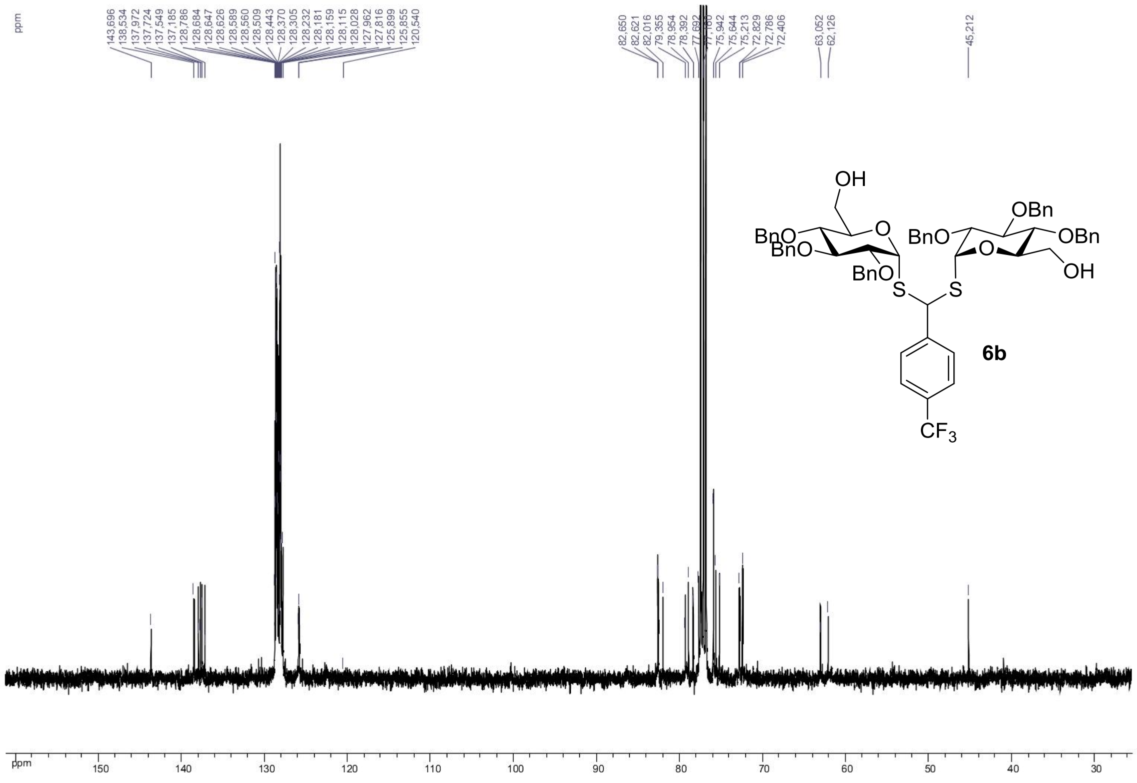

Fig 2. ^{1}H NMR (400 MHz, $CDCl_3$) and ^{13}C (100 MHz, $CDCl_3$) spectra of compound **6b**.

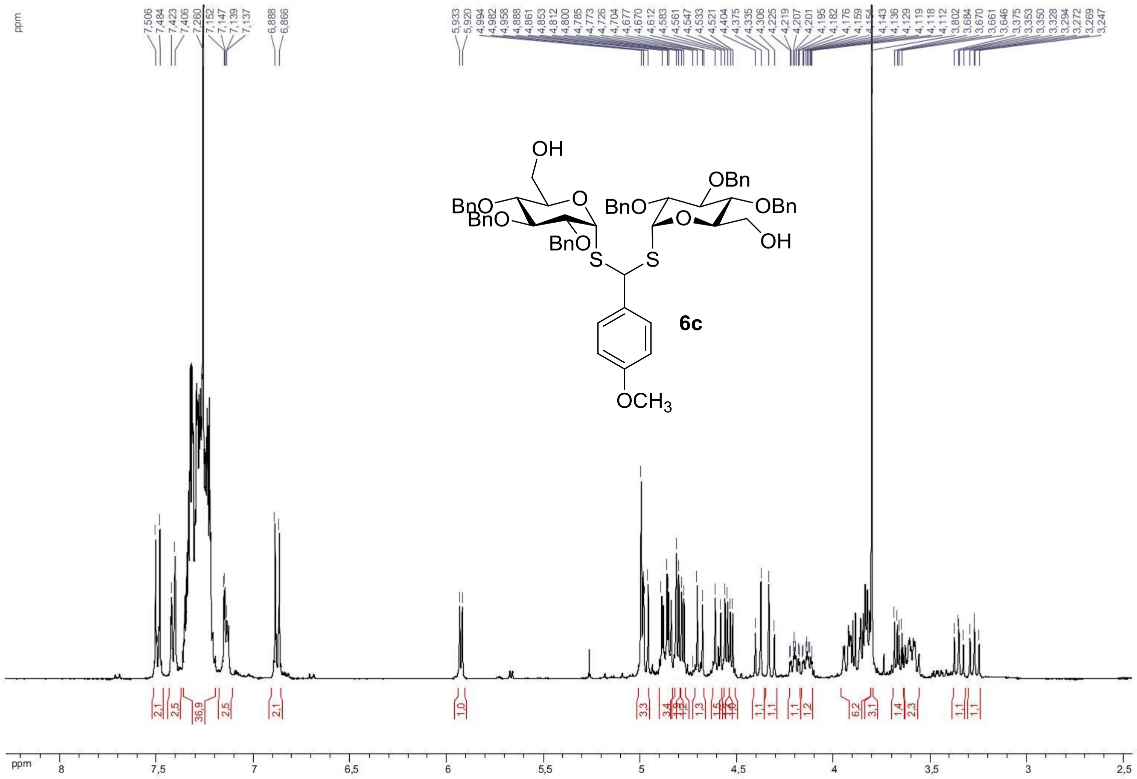

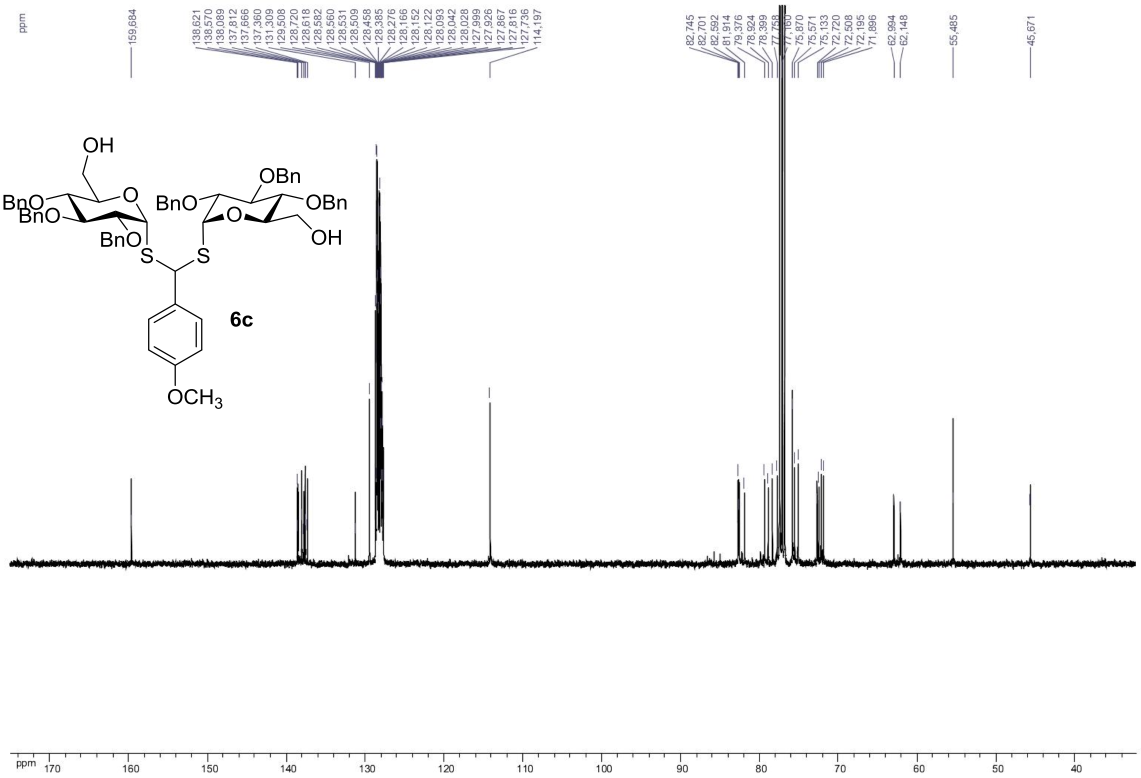

Fig 3. ^{1}H NMR (400 MHz, $CDCl_3$) and ^{13}C (100 MHz, $CDCl_3$) spectra of compound **6c**.

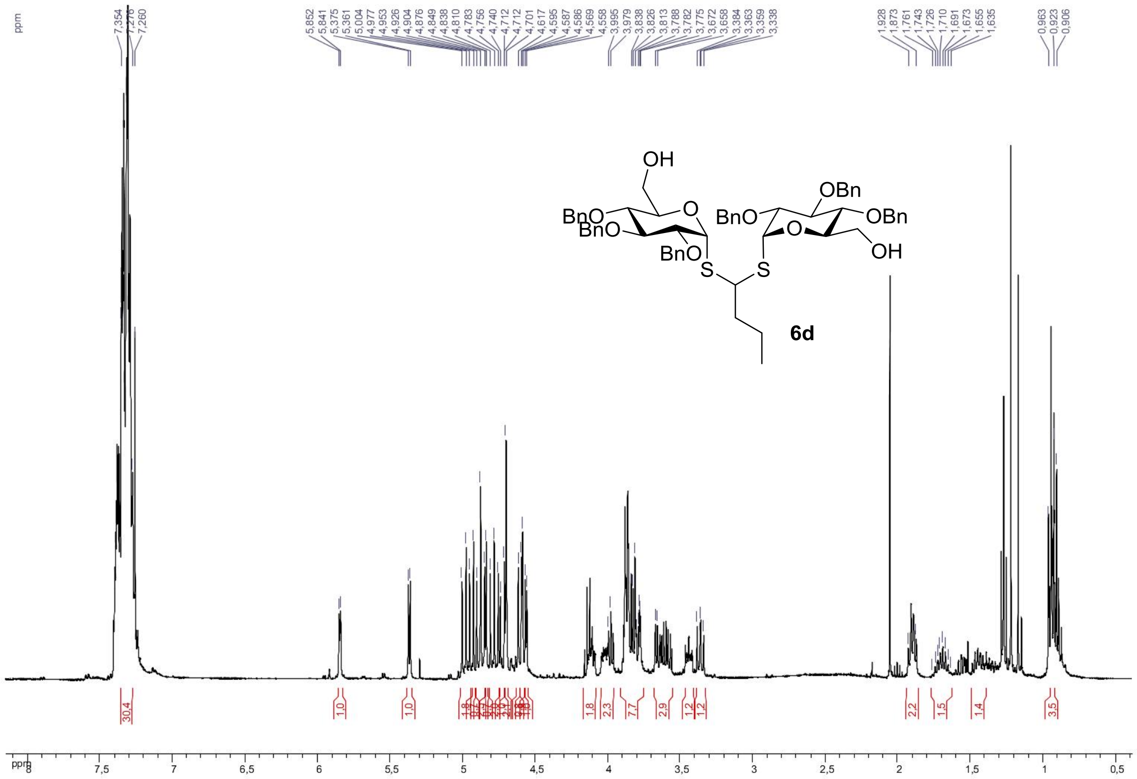

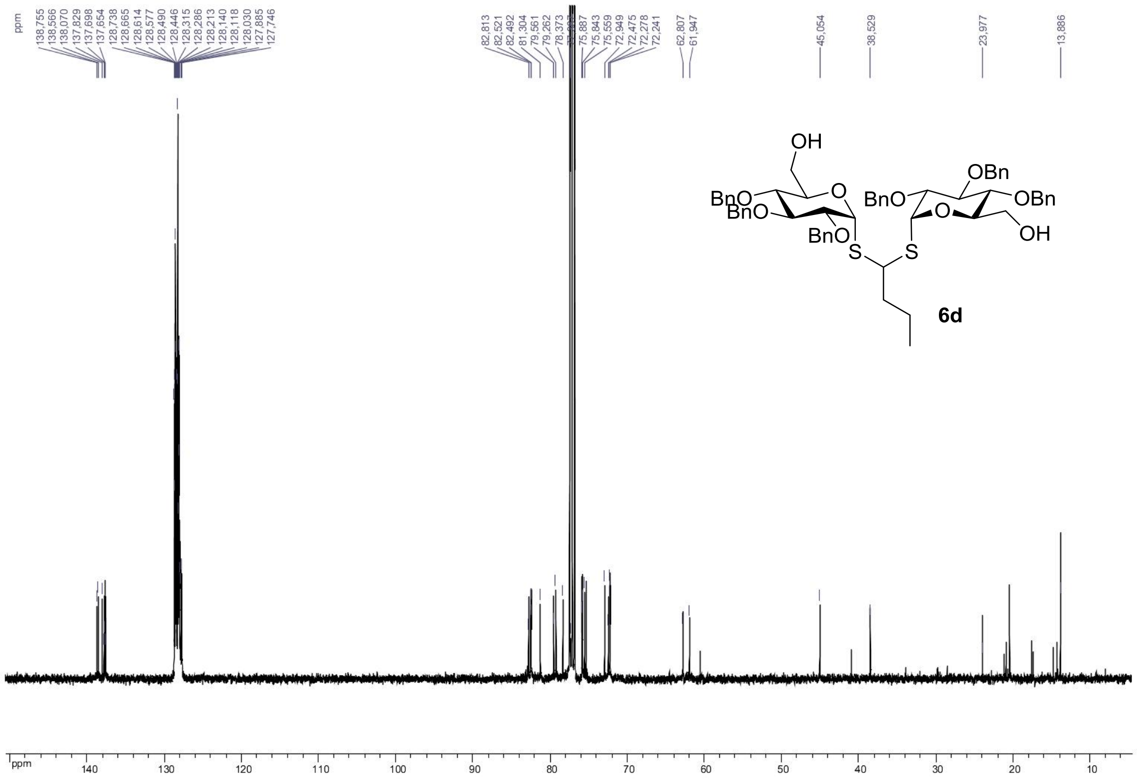

Fig 4. ^{1}H NMR (400 MHz, $CDCl_3$) and ^{13}C (100 MHz, $CDCl_3$) spectra of compound **6d**.

.

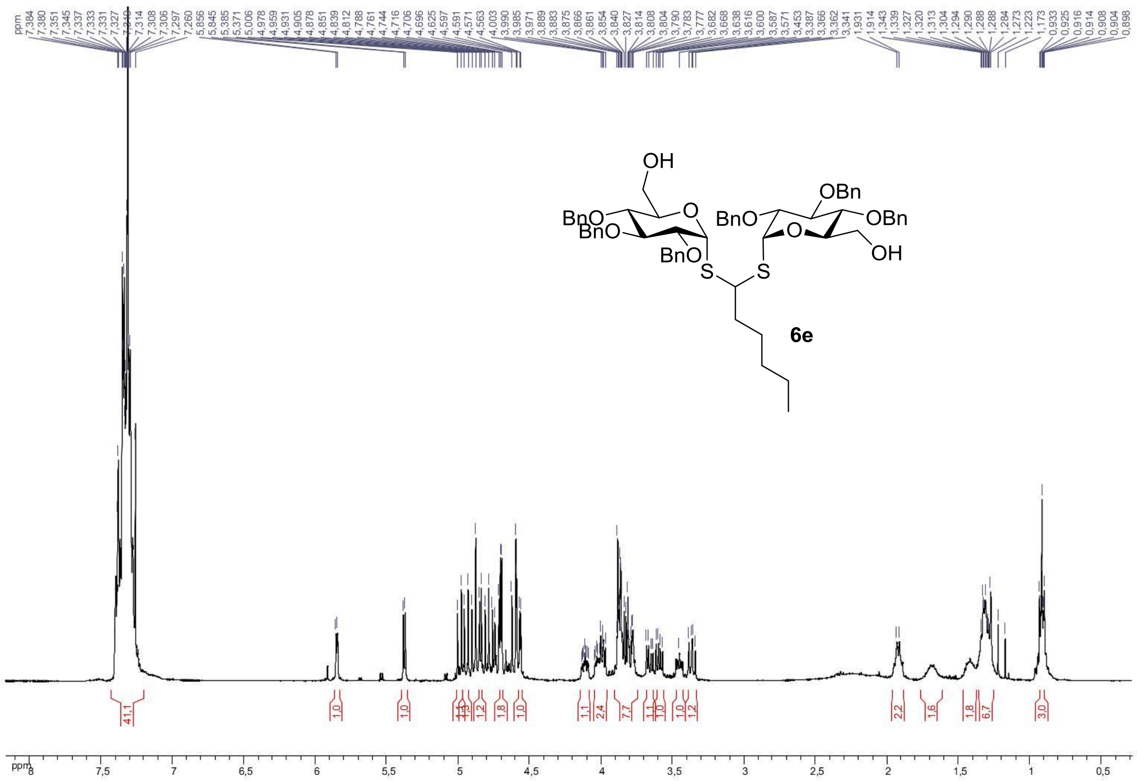

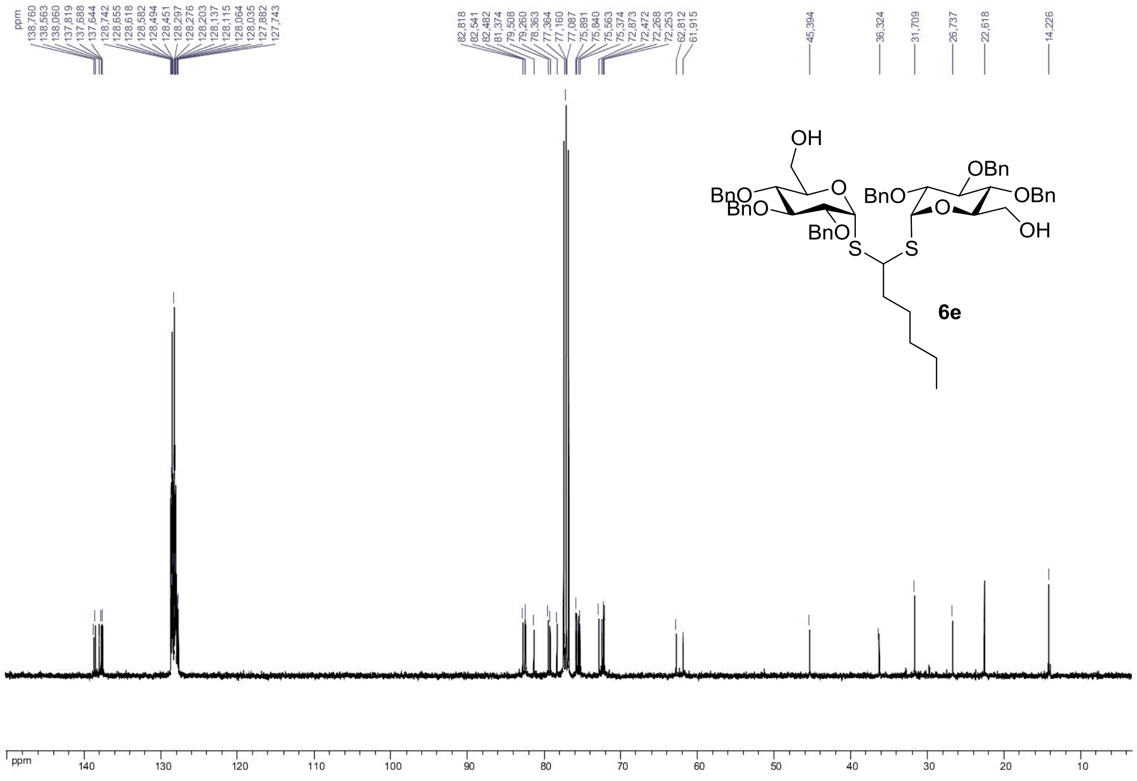

Fig 5. ^{1}H NMR (400 MHz, $CDCl_3$) and ^{13}C (100 MHz, $CDCl_3$) spectra of compound **6d**.

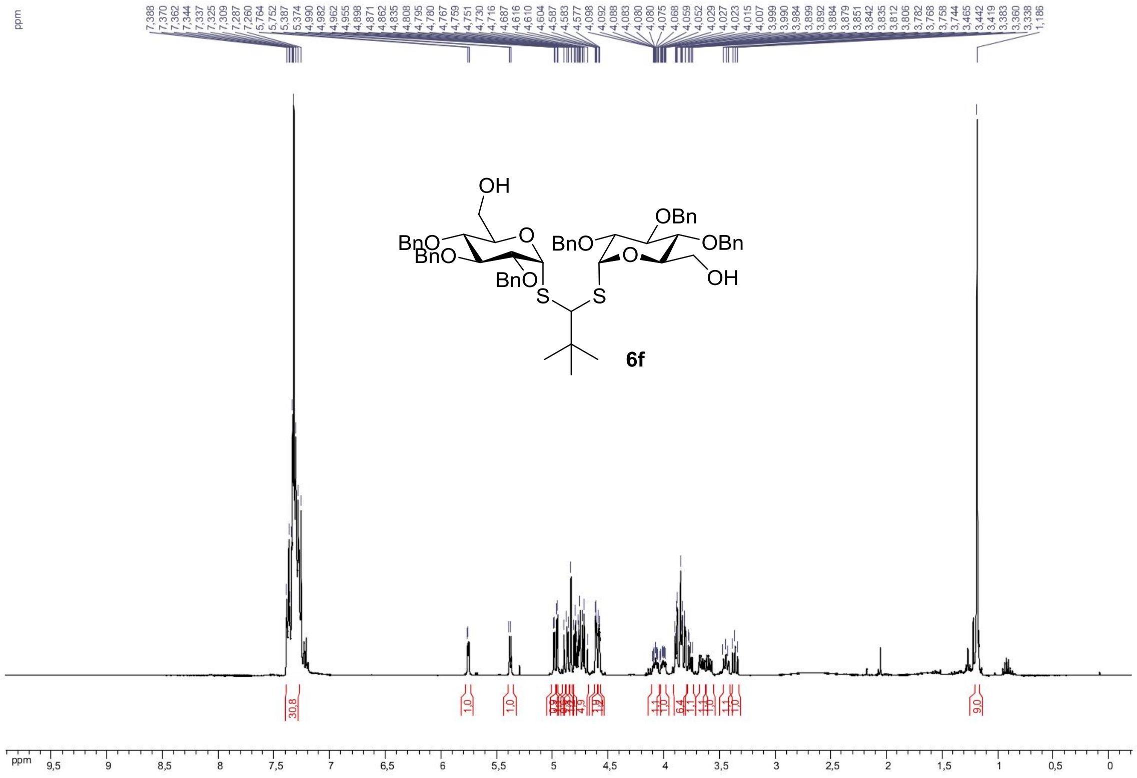

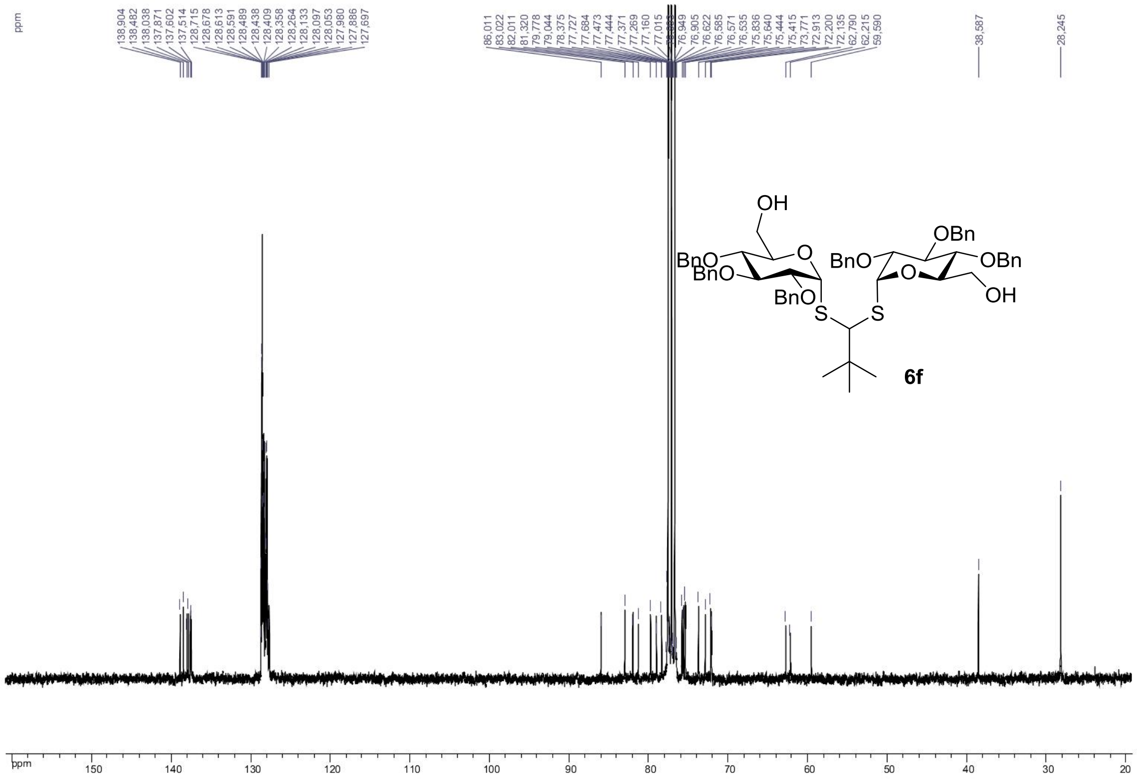

Fig 6. ^{1}H NMR (400 MHz, $CDCl_3$) and ^{13}C (100 MHz, $CDCl_3$) spectra of compound **6f**.

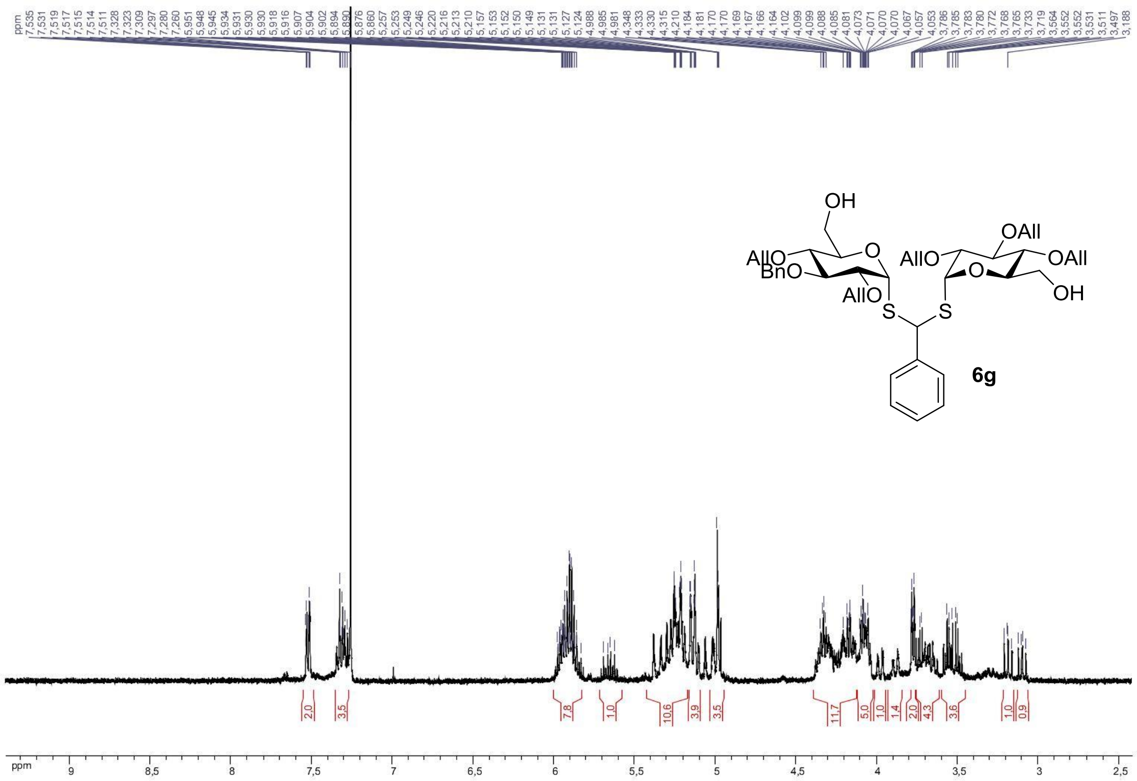

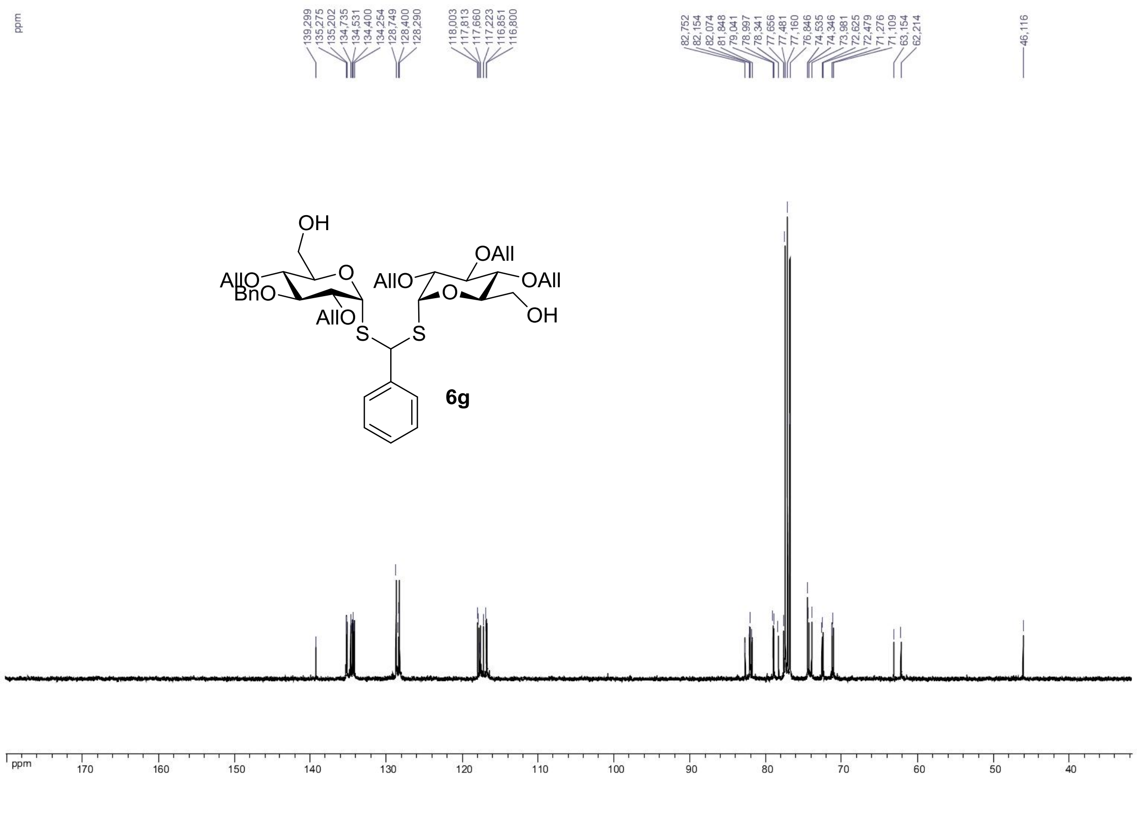

Fig 7. ^{1}H NMR (400 MHz, $CDCl_3$) and ^{13}C (100 MHz, $CDCl_3$) spectra of compound **6g**.

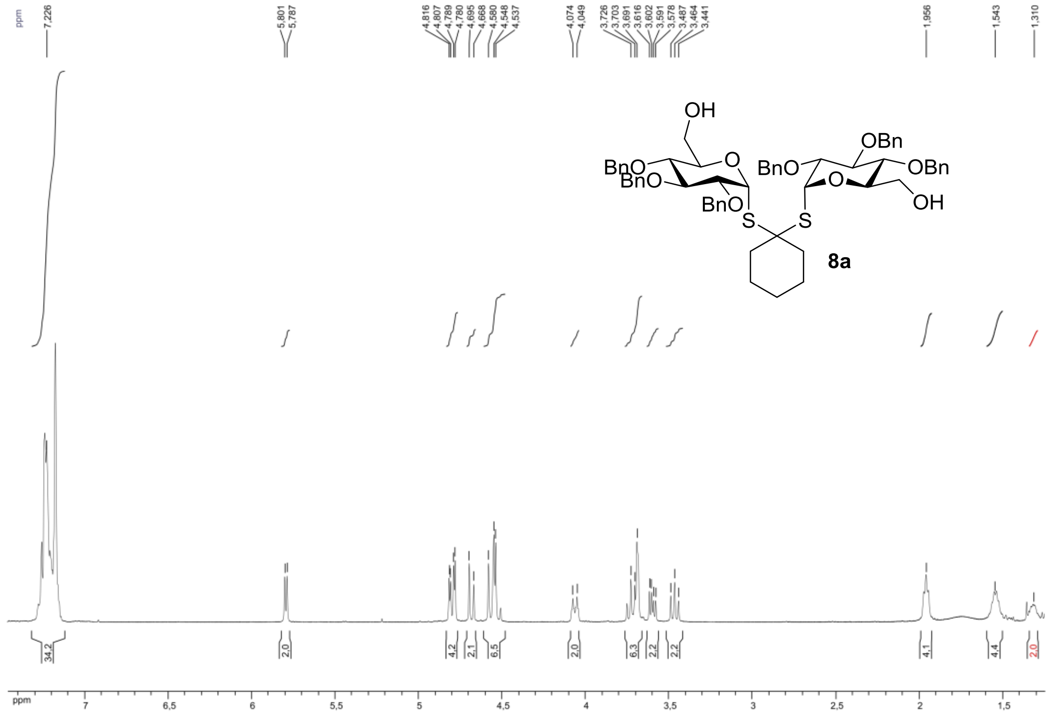

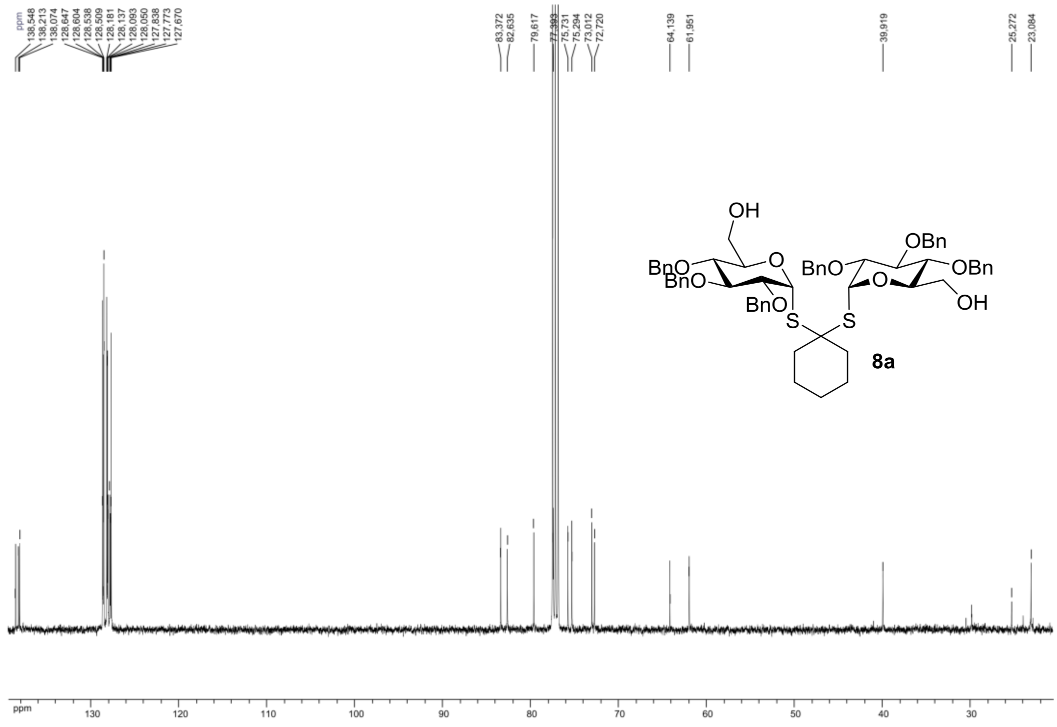

Fig 8. ^{1}H NMR (400 MHz, $CDCl_3$) and ^{13}C (100 MHz, $CDCl_3$) spectra of compound **8a**.

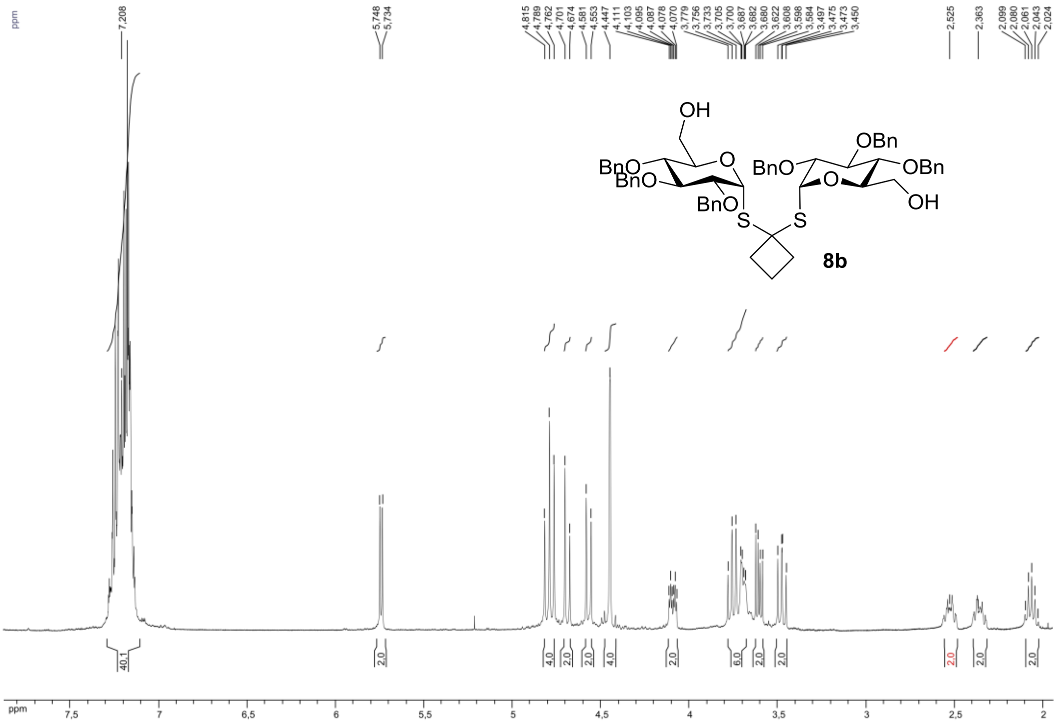

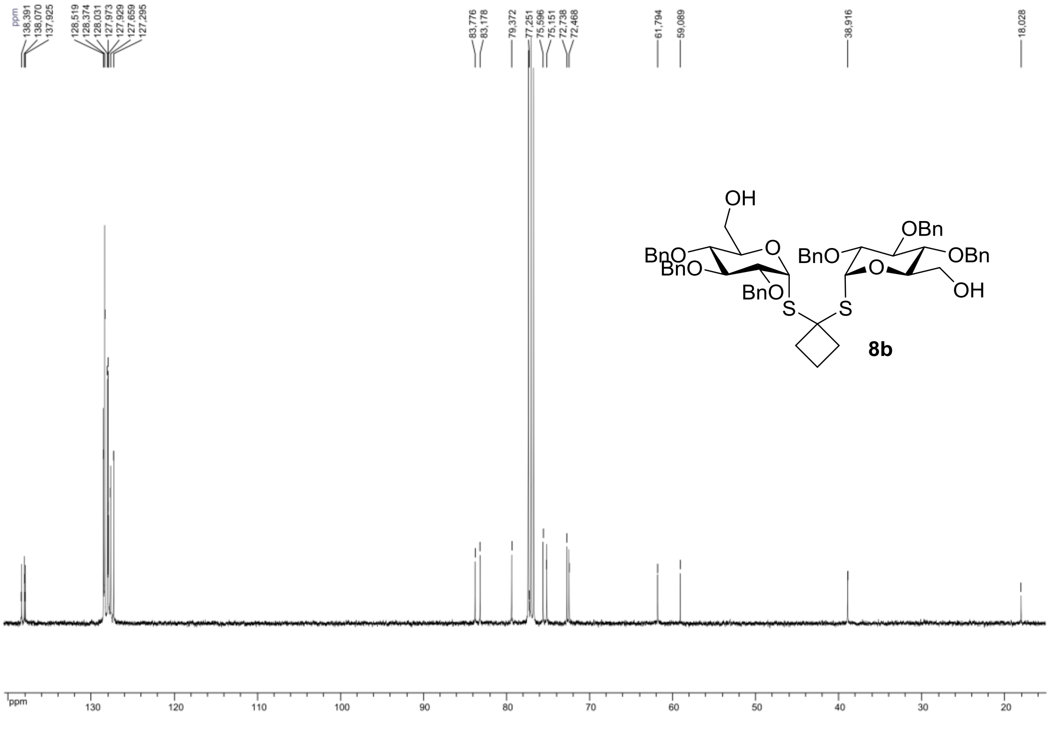

Fig 9. ^{1}H NMR (400 MHz, $CDCl_3$) and ^{13}C (100 MHz, $CDCl_3$) spectra of compound **8b**.

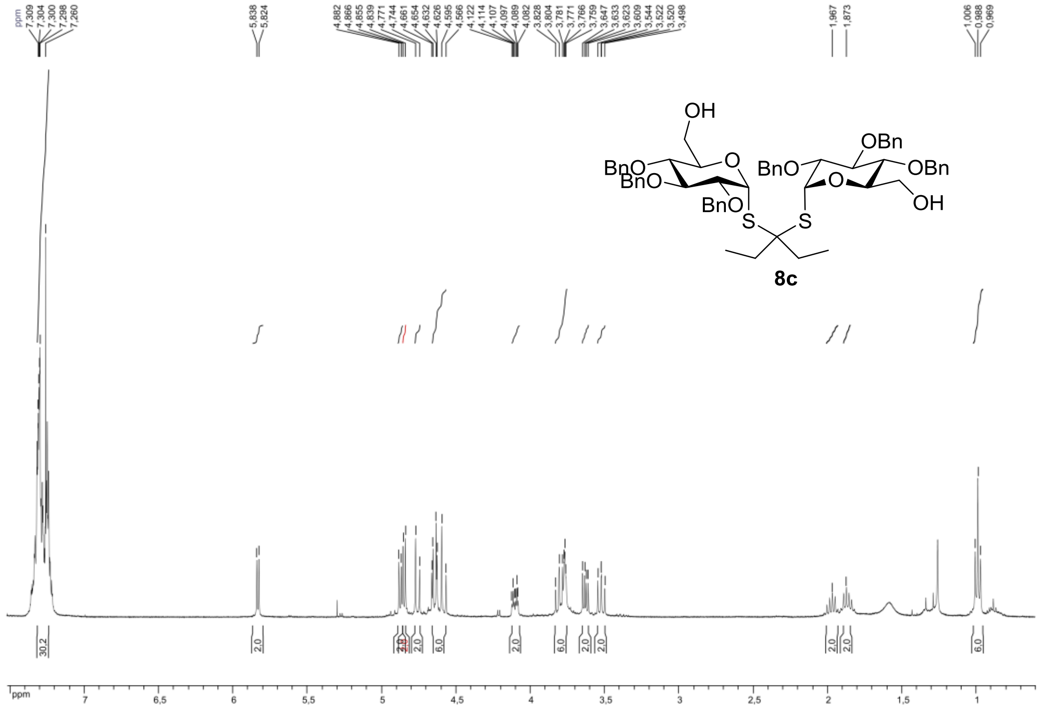

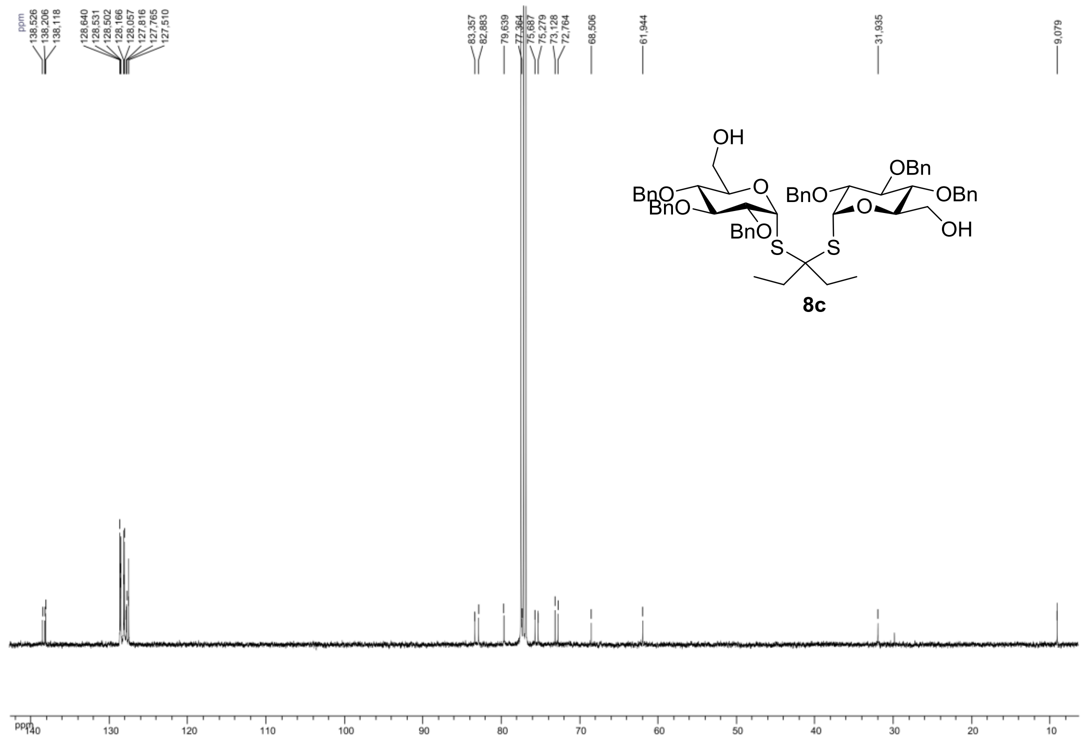

Fig 10. ^{1}H NMR (400 MHz, $CDCl_3$) and ^{13}C (100 MHz, $CDCl_3$) spectra of compound **8c**.

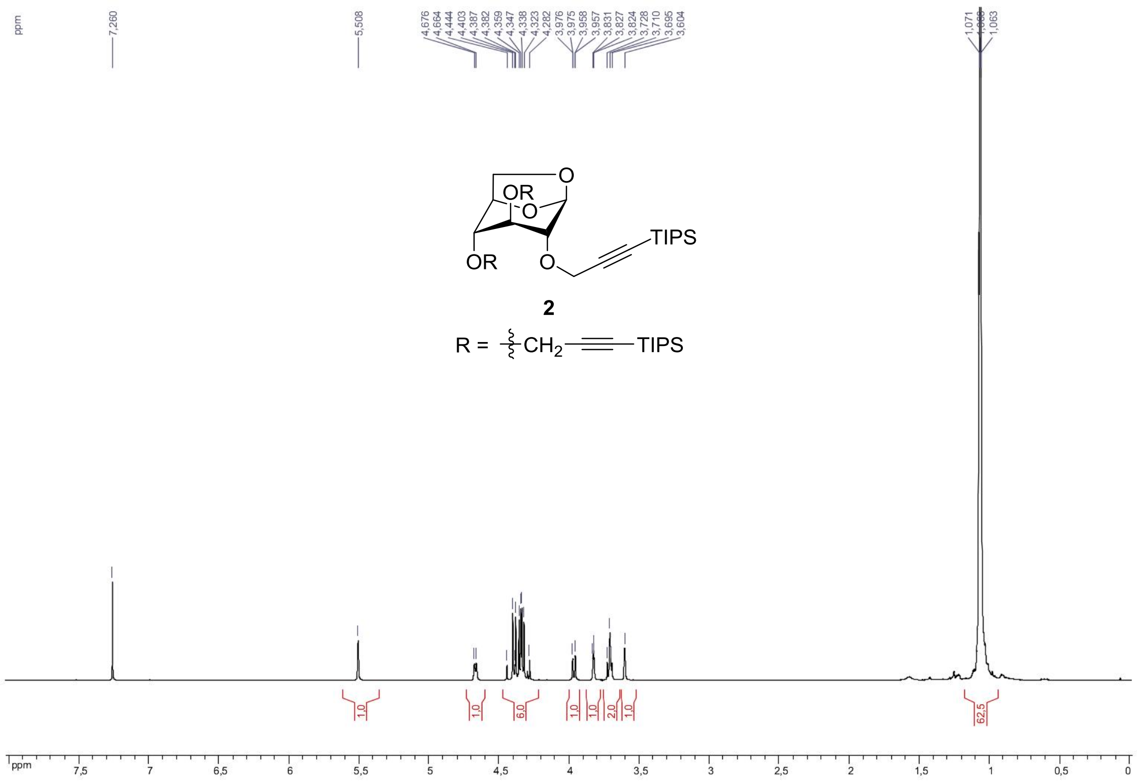

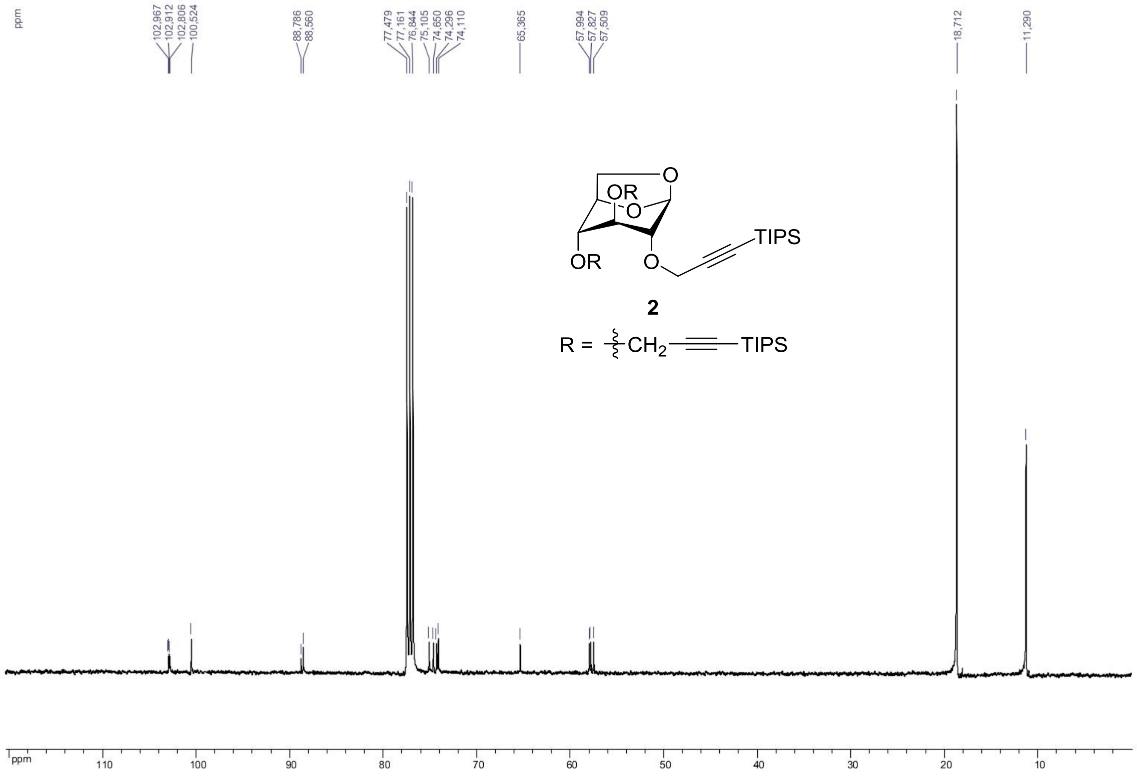

Fig 11. ^{1}H NMR (400 MHz, $CDCl_3$) and ^{13}C (100 MHz, $CDCl_3$) spectra of compound **2**.

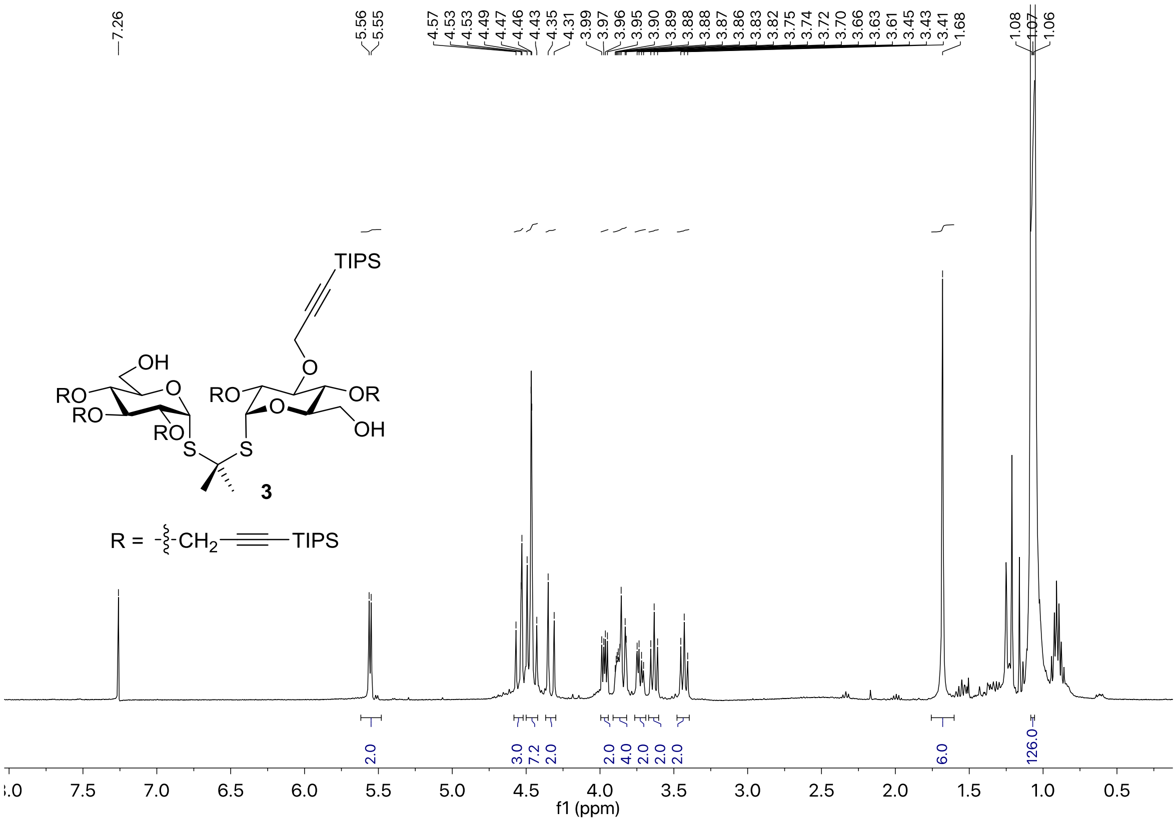

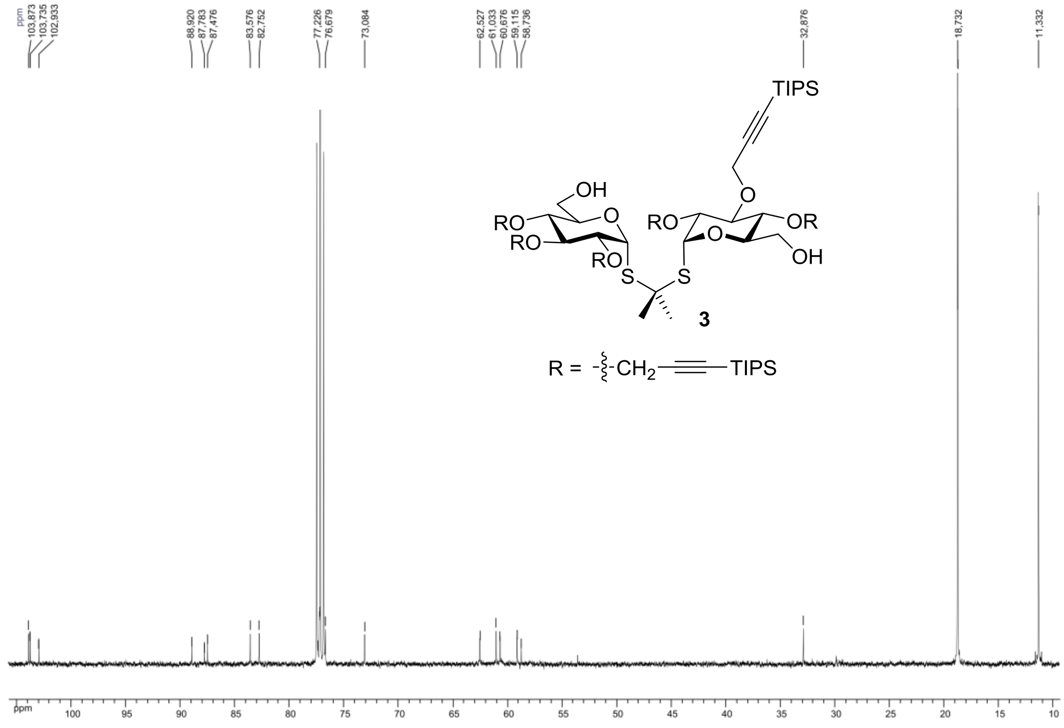

Fig 12. ^{1}H NMR (400 MHz, $CDCl_3$) and ^{13}C (100 MHz, $CDCl_3$) spectra of compound **3**.
